# Resistance to Nucleotide Excision Repair of Bulky Guanine Adducts Opposite Abasic Sites in DNA Duplexes and Relationships between Structure and Function

**DOI:** 10.1371/journal.pone.0137124

**Published:** 2015-09-04

**Authors:** Zhi Liu, Shuang Ding, Konstantin Kropachev, Jia Lei, Shantu Amin, Suse Broyde, Nicholas E. Geacintov

**Affiliations:** 1 Department of Chemistry, New York University, New York, New York, United States of America; 2 Department of Biology, New York University, New York, New York, United States of America; 3 Department of Pharmacology, Pennsylvania State University, Hershey, Pennsylvania, United States of America; University of Iowa, UNITED STATES

## Abstract

The nucleotide excision repair of certain bulky DNA lesions is abrogated in some specific non-canonical DNA base sequence contexts, while the removal of the same lesions by the nucleotide excision repair mechanism is efficient in duplexes in which all base pairs are complementary. Here we show that the nucleotide excision repair activity in human cell extracts is moderate-to-high in the case of two stereoisomeric DNA lesions derived from the pro-carcinogen benzo[*a*]pyrene (*cis*- and *trans*-B[*a*]P-*N* ^2^-dG adducts) in a normal DNA duplex. By contrast, the nucleotide excision repair activity is completely abrogated when the canonical cytosine base opposite the B[*a*]P-dG adducts is replaced by an abasic site in duplex DNA. However, base excision repair of the abasic site persists. In order to understand the structural origins of these striking phenomena, we used NMR and molecular spectroscopy techniques to evaluate the conformational features of 11mer DNA duplexes containing these B[*a*]P-dG lesions opposite abasic sites. Our results show that in these duplexes containing the clustered lesions, both B[*a*]P-dG adducts adopt base-displaced intercalated conformations, with the B[*a*]P aromatic rings intercalated into the DNA helix. To explain the persistence of base excision repair in the face of the opposed bulky B[*a*]P ring system, molecular modeling results suggest how the APE1 base excision repair endonuclease, that excises abasic lesions, can bind productively even with the *trans*-B[*a*]P-dG positioned opposite the abasic site. We hypothesize that the nucleotide excision repair resistance is fostered by local B[*a*]P residue—DNA base stacking interactions at the abasic sites, that are facilitated by the absence of the cytosine partner base in the complementary strand. More broadly, this study sets the stage for elucidating the interplay between base excision and nucleotide excision repair in processing different types of clustered DNA lesions that are substrates of nucleotide excision repair or base excision repair mechanisms.

## Introduction

Eukaryotic nucleotide excision repair (NER) is an important mammalian defense system against DNA lesions derived from environmental genotoxic agents that include polycyclic aromatic hydrocarbons (PAH). The NER process involves ~ 30 proteins and entails the excision of a 24–30-mer oligonucleotide that contains the lesion, followed by repair synthesis of the resulting gap [1]. A puzzling aspect of the NER process is the large range in the relative excision efficiencies of different DNA lesions, which vary over more than two orders of magnitude [2–4]. The complex multi-step human NER mechanism requires the recognition of the DNA damage which is a two-step mechanism. The heterodimeric XPC-RAD23B protein XPC is the crucial initial sensor of disturbances in the normal structure of double-stranded DNA that binds to the DNA damage and recruits the next multiprotein factor TFIIH. The subsequent step verifies that a damaged DNA base is actually present [5, 6] by a mechanism that involves the activity of the helicase XPD [7]. The XPC-RAD23B—DNA complex stimulates the recruitment of subsequent NER factors [5, 8], which ultimately leads to the characteristic dual incisions of the damaged strand that yield the single-stranded, 24–32 nucleotide-long DNA sequence that contains the lesion. In human cell extracts, the yields of dual incision products depends on factors that include: (1) the base sequence context in which the DNA lesions are embedded [9–11], (2) the presence or absence of the Watson-Crick partner nucleotides opposite the modified nucleotide [12], and (3) the identity of a mismatched base opposite the modified nucleotide [13].

**Fig 1:**
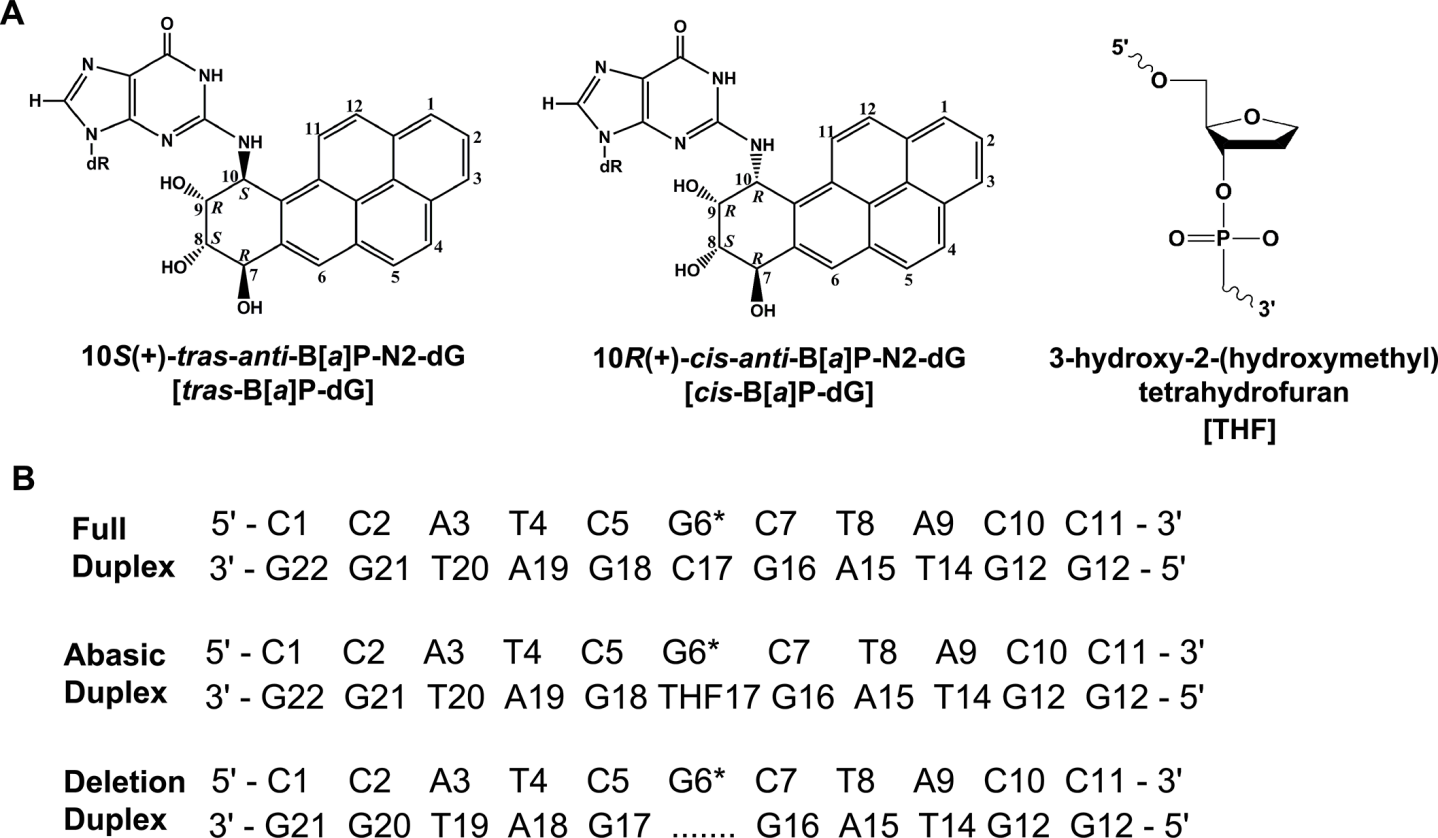
Chemical structures of the lesions and sequences. (A) Chemical structures of the *trans*-B[*a*]P-dG, *cis*-B[*a*]P-dG and the THF site. The THF is a stable analog of an abasic (AB) site. (B) The sequences and numbering system of the 11-mer duplexes containing the *trans*-B[*a*]P-dG adduct. G* denotes B[*a*]P-dG adduct.

Structure-function studies of DNA lesions derived from the prototype procarcinogen benzo[*a*]pyrene have played an important role in elucidating the features of damaged duplexes that lead to their recognition by the NER recognition protein XPC-RAD23B. The major adduct derived from the metabolic activation of benzo[*a*]pyrene through the well-characterized diol epoxide pathway [14] is the 10*S*-(+)-*trans*-B[*a*]P-dG (*trans*-B[*a*]P-dG) adduct [15] shown in Fig 1. This adduct, situated in the B-DNA minor groove in duplex DNA [16], is generally moderately well-repaired depending on sequence context [9–11], but removing the partner nucleotide in the unmodified strand opposite the *trans*-B[*a*]P-dG adduct completely abolishes the NER response in human HeLa cell extracts [17]. However, the removal of this nucleotide causes a change in the conformation of the bulky aromatic polycyclic residue, from a minor groove location in the full duplex to a base-displaced intercalated conformation in the ‘deletion’ duplex [18]. A similar phenomenon is observed in the case of the stereoisomeric 10*R*-(+)-*cis*-B[*a*]P-dG adduct (*cis*-B[*a*]P-dG) (Fig 1); this adduct exhibits a ~ five times greater NER activity than the 10S (+)-*tran*s duplex, but is also completely resistant to NER in a deletion duplex lacking the nucleotide opposite the adduct [12, 19, 20]. Thus, the nature and presence of the partner nucleotide seems to play a key role in determining NER efficiency. The importance of the partner base opposite the lesion has been underscored by the crystal structure of the yeast ortholog Rad4-Rad23B of the human XPC-RAD23B NER lesion recognition factor, in a complex with a DNA duplex containing a cyclobutane pyrimidine (CPD) T^T lesion opposite a TT mismatch in the complementary strand; this structure shows that the two bases opposite the lesion are flipped into the protein, while a BHD3 β hairpin is inserted into the duplex from the major groove at the lesion site [21]. The interactions of these two flipped out bases with aromatic and hydrophobic amino acid residues stabilize this DNA—protein complex.

The abrogation of NER activity in deletion duplexes that lack the nucleotide opposite the B[*a*]P-dG adduct has been attributed to extensive stacking interactions between the intercalated polycyclic aromatic ring system and the adjacent base pairs, so that the insertion of the β-hairpin is hindered [10, 12, 18, 22] and NER activity is abolished [12, 19]. In order to elucidate how features of the local DNA binding site and adduct conformation affect NER activity, we explored the impact of positioning the *trans-*B[*a*]P-dG adduct opposite a tertrahydrofuran (THF) abasic site (AB); the AB site lacks only the cytosine base in the complementary strand opposite the modified guanine, but not its 2’-deoxyribose residue and phosphodiester group (Fig 1). The conformation and NER susceptibility of the *trans*-B[*a*]P-dG adduct opposite an AB site are compared to the structural characteristics and NER response of the same adduct in a full duplex and manifests moderate NER response in the same sequence [19, 23]. The impact of stereochemistry are examined by evaluating the adduct conformation and NER activity of the stereoisomeric *cis*-B[*a*]P-dG adduct (Fig 1). Using NMR methods, it is shown that the *trans*-B[*a*]P-dG adduct assumes a base-displaced intercalated conformation when it is positioned opposite an AB site, in contrast to its minor groove conformation in the full duplex [24]. However, its NER activity is eliminated when positioned opposite an AB site. A similar abrogation of the NER activity is observed in the case of the *cis-*B[*a*]P-dG adduct which is an excellent NER substrate in the full duplex, and which is likewise intercalated in both the full and AB duplexes.

## Methods

### Preparation and thermodynamic stabilities of the *trans*-B[*a*]P-dG:AB 11-mer duplexes

The single-stranded, site-specifically modified 11-mers, 5’-CCATCG*CTACC with a *trans*-B[*a*]P-dG lesion at the central G* were synthesized by a direct method as described previously [24–26]. This strand was annealed to the complementary strand containing the AB (THF) abasic site opposite G6* to generate the *trans*-B[*a*]P-dG:AB duplex. The thermodynamic stabilities of these duplexes were measured by determining the melting points (T_m_) of the 11/11-mer duplexes. The T_m_ values, defined as the temperature at which 50% of the duplexes are dissociated, were determined by monitoring the absorbance of DNA duplexes at 260 nm as a function of increasing temperature from ~ 15 to 65–70 ^0^C under standard conditions in 20 mM sodium phosphate buffer solution, pH 7.0, 100 mM NaCl, 20 μM DNA duplex concentration.

This duplex was used in the new NMR structural studies reported here, and the base numbering system is defined in Fig 1. The sequences of the ‘full’ duplex with all 11 base pairs intact, as well the sequence complementary to the modified strand in the ‘deletion’ duplex are also defined.

### NMR measurements

Through-space nuclear Overhauser effects and through-bond correlated 2D spectra were recorded and analyzed in order to assign the B[*a*]P and nucleic acid protons in the *trans*-B[*a*]P-dG:AB abasic 11-mer duplex. To visualize the imino protons, 2D nuclear Overhauser effect spectroscopy (NOESY) data for the modified duplex in 10% H_2_O buffer (100 mM NaCl, 10 mM phosphate, pH 6.8) at 10 ^0^C were recorded; a mixing time of 200 ms employing a jump-return pulse sequence for solvent suppression on a Bruker 500 Mhz NMR spectrometer equipped with a TXI probe was utilized. The corresponding NOESY spectra in D_2_O buffer at 10°C were recorded at a mixing time of 300 ms. Through-bond correlation spectroscopy (COSY) and total correlation spectroscopy (TOCSY) at a 300 ms mixing time were recorded using a Bruker 800 MHz NMR instrument equipped with a cryoprobe at the New York Structural Biology Center (NYSBC). Peak assignments were obtained using the SPARKY program [27].

### Restrained MD simulations

The NMR data indicated that the *trans*-B[*a*]P-dG adduct opposite a THF AB site adopts a base-displaced intercalated conformation in the 11-mer duplex with the THF moiety opposite the lesion. We utilized an equilibrated initial model of this duplex (see S1 Methods for details) for NMR distance-restrained MD simulations of 1 ns. Table 1 lists the experimental NOE restraints that were employed. Weights for the NOE and Watson-Crick base pair restraints were in the range of 5–20 kcal/mol·Å. The following distance restraint ranges were utilized: strong NOEs, 2.5–3.5 Å; medium strength NOEs, 3.5–4.5 Å, and weak NOEs, 4.5–6.0 Å. The computations were carried out using the AMBER 9 simulation package [28], the Cornell et al. force field [29] with the parm99.dat parameter set [30]. Details of the force field and the MD simulation protocol are given in S1 Methods. The force field parameters for the THF and the *trans*-B[*a*]P-dG adduct are given in S1 and S2 Tables. We collected 1000 structures, taken at 1 ps intervals, from the 1 ns restrained MD simulation. To represent the NMR data, we obtained five representative structures using clustering by the RMSD metric with the K-means algorithm [31] in the Ptraj module of AMBER.

**Table 1:** Observed intermolecular NOEs in the *trans*-B[*a*]P-dG:AB duplex and achieved distances from the representative structures in the restrained MD simulation.*^a^*

B[*a*]P Proton	Chemical Shift (ppm)	NOE	Intensity^*a*^	Achieved Distances (Å)
H1	7.500	C5 (H1')	W	5.0 (0.2)
H2	7.250	G18 (H4')	W	6.0 (0.1)
		THF17(H2'1)	W	4.7 (0.2)
		THF17(H2'2)	W	5.6 (0.2)
H3	6.820	THF17(H2'1)	W	4.3 (0.2)
		THF17(H2'2)	W	4.8 (0.2)
		G18 (H5'2)	S	3.5 (0.3)
H4	6.636	G16 (H1')	M	4.1 (0.3)
		G16 (H2'1)	W	5.2 (0.2)
		G16 (H2'2)	W	6.3 (0.1)
		G18 (H4')	W	4.3 (0.1)
		G18 (H3')	W	5.4 (0.0)
		G18 (H5'2)	S	2.4 (0.0)
		THF17(H2'1)	W	4.9 (0.1)
		THF17(H2'2)	W	4.5 (0.1)
H5	6.660	G16 (H1')	M	3.7 (0.4)
		G16 (H2'1)	W	4.2 (0.1)
		G16 (H2'2)	W	4.8 (0.1)
		G18 (H4')	W	6.3 (0.1)
		G18 (H3')	Very W	6.6 (0.1)
		G18 (H5'2)	S	3.7 (0.1)
		THF17(H2'1)	W	6.3 (0.1)
		THF17(H2'2)	W	5.3 (0.2)
H6	7.237	G16 (H1')	M	4.6 (0.1)
H7	4.831			
H8	4.022			
H9	4.112			
H10	6.603	C7 (H5)	W	4.9 (0.3)
H11	8.303	C7 (H5)	W	4.4 (0.1)
H12	7.039	C5 (H1’)	M-S	4.1 (0.2)
		C5 (H2'1)	M	4.2 (0.2)
		C5 (H2’2)	W	5.6 (0.2)
		C7 (H5)	M	4.5 (0.1)

^*a*^ Experimentally observed NOEs were employed as restraint bounds in the distance restrained MD simulation. The bounds assigned based on the observed intensities are: [S] = 2.5–3.5 Å [M] = 3.5–4.5 Å, [W] = 4.5–6.0 Å, [M-W] = 3.5–6.0 Å. S = strong, M = medium, W = weak.

### Structural Analyses

The PTRAJ module of AMBER 9 was employed for structural analyses. The INSIGHTII program (Accelrys Software, Inc.) was employed for visualizing and model building. The figures and movies were prepared with Pymol [32].

### Preparation of the B[*a*]P-dG adduct-containing 135-mer DNA duplexes

The NER assays were conducted with 135-mer DNA duplexes that were constructed by ligation methods using the 11-mer sequence defined in Fig 1, employing T4 ligase (USB Molecular Biology Reagents and Biochemicals, Cleveland, OH) as described elsewhere [19]. The 11-mers were ^32^P-endlabeled before ligation so that the 24–30-mer dual incision products could be visualized after separation of the dual incision fragments from the 135-mer duplexes by gel electrophoresis methods. The B[*a*]P-dG adduct opposite the abasic site was positioned at the 68^th^ nucleotide counted from the 5’-side, in the middle of the 135-mer duplex. Briefly, the 11-mer oligonucleotide 5'-d(CCATCG*CTACC) was 5'-end labeled with [γ-^32^P]ATP (6000 Ci/mmol, PerkinElmer Life Sciences, Boston, MA) and incorporated into a 135-mer duplex using T4 ligase as described previously [11, 19] These internally and radioactively labeled 135-mers were purified using 12% denaturing polyacrylamide gels and were subsequently annealed with their fully complementary 135-mer strands by heating the solutions to ~ 90°C for 2 min and cooling overnight to 4°C. The same annealing procedure was employed to synthesize fully complementary duplexes with C in the complementary strand opposite G*. A similar full duplex containing a cisplatin intrastrand crosslink in the 5’-CTCTTCTTCTG*TG*CA sequence context in otherwise identical 135-mer duplexes (the asterisks (*) denote the sites of attachment of the Pt atom) were synthesized [23], and used as positive NER controls.

### NER assay experiments

The HeLa cell extracts were prepared using standard methods [33] that were slightly modified as described [34]. The ^32^P-labeled 135-mer duplexes were incubated in 80 μL aliquots of the cell extracts (containing 60–80 μg of protein) for time intervals of up to 30 minutes. The oligonucleotide excision products and intact DNA were desalted by precipitation with an aqueous 80% methanol solution and subjected to denaturing 12% polyacrylamide gel electrophoresis. The dried gels were then analyzed by gel autoradiography using a Storm 840 phosphorimager.

## Results

### NMR characterization of the *trans*-B[*a*]P-dG:AB duplex

The bulky aromatic ring system of the *trans*-B[*a*]P-dG adduct in a full duplex, with all base pairs intact, assumes a minor groove conformation with a 5’-orientation relative to the modified guanine base [24]. However, we show here that removing only one base from the duplex, the base C opposite the modified guanine residue G6*, while leaving intact the 2’-deoxyribosyl residue and phosphodiester group, results in a striking change to a base-displaced intercalated conformation. The structure of the 11-mer duplex containing the *trans*-B[*a*]P-dG adduct in the oligonucleotide 5’-C1-C2-A3-T4-C5-G6*-C7-T8-A9-C10-C11 at position G6* (Fig 1), annealed with the partner strand containing a THF site opposite G6*, was obtained by a combination of NMR and computational methods.

Following previously established procedures, the assignment of the majority of the chemical shifts was based on TOCSY (75ms mixing time) and NOESY (300 ms mixing time with solutions of different concentrations) data sets, and then confirmed by water NOESY (250 ms mixing time) and COSY methods. These results are summarized in S3 Table. Analogous data for the unmodified duplex is shown in S4 Table for comparison.

#### Exchangeable proton spectra

**Fig 2:**
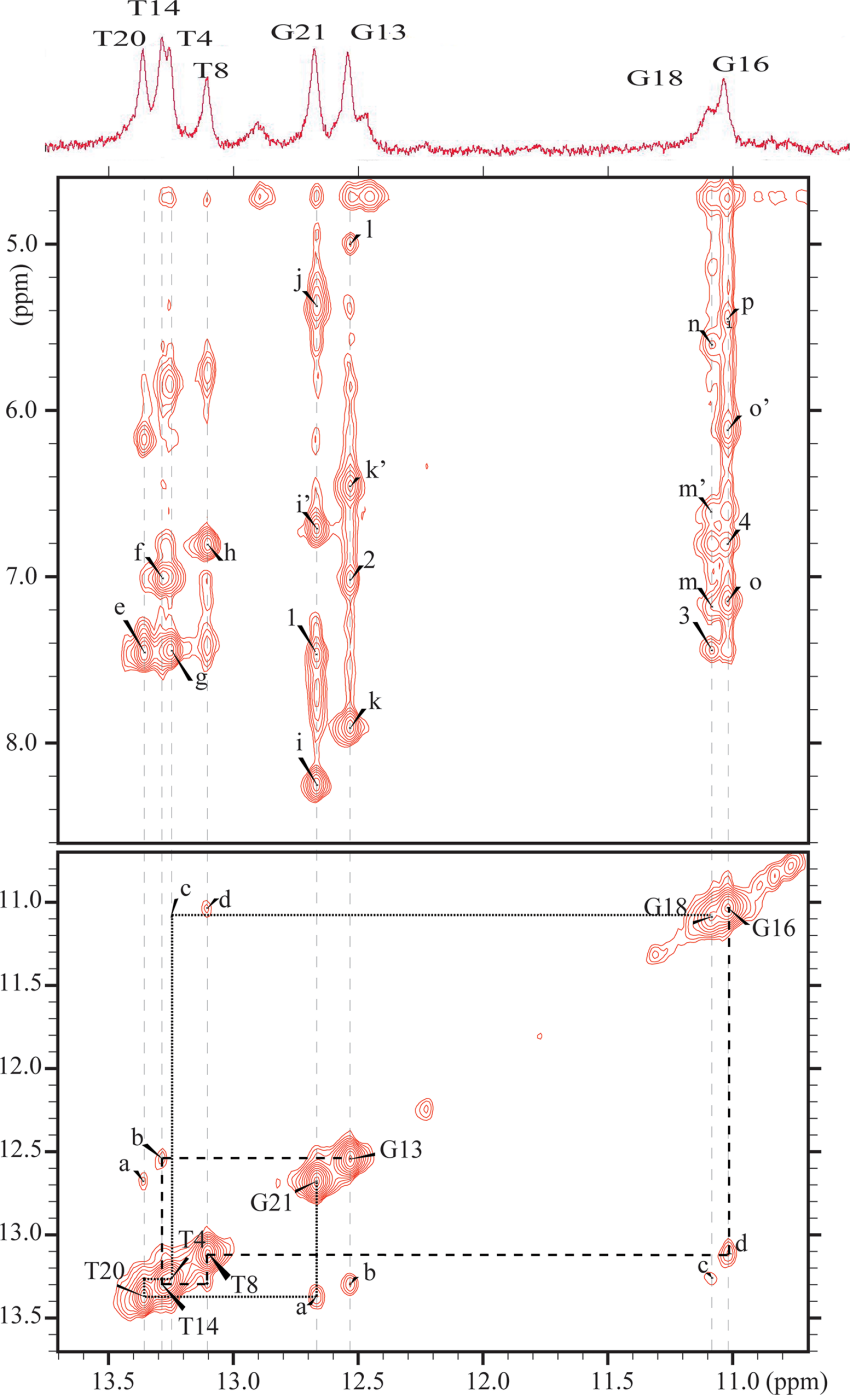
1D and 2D NMR spectra characteristics of the *trans*-B[*a*]P-dG:AB duplex. (A) 1D spectrum (10.5–14 ppm) showing the imino proton assignments. (B) Portion of a 2D NOESY (250 ms mixing time) contour plot recorded at 10°C, in 10% H_2_O solution showing NOE connectivities between amino (5–8.5 ppm) and imino protons (10.8–13.8 ppm), and (C) imino- imino protons (10.8–13.8 ppm). Assignments: a, T20(NH3)—G21(NH1); b, T14(NH3)—G13(NH1); c, T4(NH3)—G18(NH1); d, T8(NH3)—G16(NH1); e, A3(H2)—T20(NH3); f, A9(H2)—T14(NH3); g, A19(H2)—T4(NH3); h, A15(H2)—T8(NH3); i,i’, C2(NH,H’)—G21(NH1); j, C2(H5)—G21(NH1); k, k’, C10(NH,H’)—G13(NH1); *l*, C10(H5)—G13(NH1); m, m’, C5(NH,H’)—G18(NH1); n, C5(H5)—G18(NH1); o, o’, C7(NH,H’)—G16(NH1); p, C7(H5)—G(NH1); q, A3(H2)—G21(NH1); r, A9(H2)—G13(NH1); s, A19(H2)—G18(NH1); t, A15(H2)—G16(NH1).

The 1D exchangeable NMR spectrum of the *trans*-B[*a*]P-dG:AB duplex in 10% H_2_O buffer, pH 6.8 at 10°C is plotted in Fig 2A; the imino proton assignments are also shown using the numbering system defined in Fig 1C. The partially resolved imino proton resonances between 12 and 13.5 ppm are characteristic of Watson-Crick base pairing for short DNA duplexes. In addition, two other, partially resolved imino proton resonances that are upfield-shifted to 11.02 and 11.09 ppm have been observed in the case of other adducts with intercalative B[*a*]P-dG adducts [22, 35] and are assigned to the imino protons flanking the adduct site, G16 and G18. These up-field shifted imino proton resonances are due to ring current effects associated with the intercalated pyrenyl aromatic ring system of the *trans*-B[*a*]P-dG adduct, positioned inside the helix between G16 and G18. The absence of the dG6* imino proton resonance suggests that this guanine imino proton may be looped out of the helix and is exposed to solvent where it undergoes rapid exchange with water. The imino proton signals of the terminal base pairs are not detectable because of fraying effects that are also characterized by rapid exchange with solvent.

**Fig 3:**
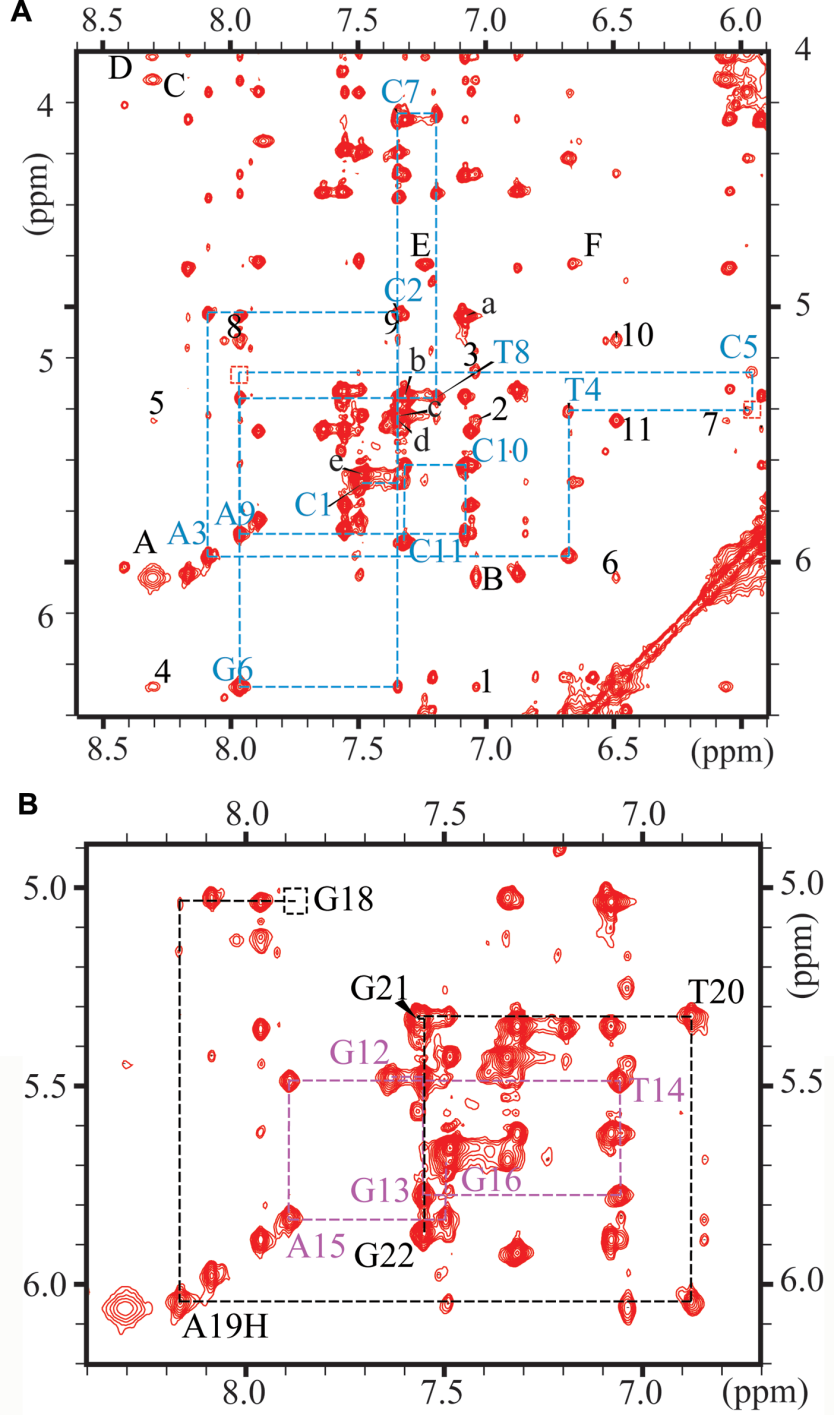
Expanded contour plot of a NOESY spectrum (300 ms mixing time) of the *trans-*B[*a*]P-dG:AB duplex. The spectrum was obtained in D_2_O aqueous buffer solution at 10°C using an 800 MHz spectrometer equipped with a cryoprobe. The focus is on the base (purine H8 and pyrimidine H5 and H6) and sugar H1’ proton region. (A) Sequential assignments from dC1 to dC11 on the modified strand (blue dashed line) A:B[*a*]P10-B[*a*]P11, B: B[*a*]P10-B[*a*]P12, C: B[*a*]P9-B[*a*]P11, D: B[*a*]P8-B[*a*]P11, E: B[*a*]P7-B[*a*]P6, F: B[*a*]P7-B[*a*]P5; 1: G6(H1’)-B[*a*]P12, 2: C7(H5)-B[*a*]P12, 3: C5(H1’)-B[*a*]P12, 4: G6(H1’)-B[*a*]P11, 5: C7(H5)-B[*a*]P11, 6: G6(H1’)-B[*a*]P10, 7: C7(H5)-B[*a*]P10, 8: G6(H3’)-H8, 9: G6(H3’)-C7(H6), 10: G6(H3’)-(H1’), 11: C7(H5)-G6(H1’); a: C10(H5)-(H6), b: C11(H5)-(H6), c: C2(H5)-(H6), d: C7(H5)-(H6), e: C1(H5)-(H6) (B) Sequential assignments from dG12 to dG16 (purple dashed line); from dG18 to dG22 (black dashed line) in the THF-containing complementary strand. Expanded NOESY contour plot in D_2_O buffer at 10°C establishing the NOE connectivity between base protons (purine H8 and pyrimidine H6) and their own and 5’-flanking sugar H1’ protons.

An expanded region of the water NOESY contour plot (250ms mixing time) in 10% H_2_O buffer at pH 6.8 and at 10°C is shown in Fig 2B. Strong interactions between T(N3H) and A(H2) protons (Fig 2B, peaks e, f, g, h) serve to identify all Watson-Crick A-T base pairs in the *trans*-B[*a*]P-dG:AB duplex. The A(H2) protons also show NOE interactions to their adjacent guanine imino protons (peaks q, r, s, t) which suggest base pair stacking [36] at those base pairs in the duplex. The NOESY results in D_2_O buffer (Fig 3) confirm the identification of the A(H2) protons to be in the range shown in Fig 2B. Similarly strong NOE peaks between the G(N1H) imino protons and the cytosine amino protons (Fig 2B, peaks i, i’, k, k’) provide further evidence for the existence of Watson-Crick hydrogen bonding except at the G16:C5 and G18:C7 base pairs. The NOE connectivities of the base pairs flanking the *trans*-B[*a*]P-dG are weaker (Fig 2B, peaks m, m’, o, o’), especially at C5(NH,H’)—G18(NH1)(m,m’).

Fig 2C shows the sequential imino to imino proton NOE connectivities between adjacent base pairs from G13-T14-T8-G16 on the 5’-side of THF, and from G18-T4-T20-G21 on its 3’-side.

The temperature-dependence of the imino proton spectrum is shown in S1 Fig A comparison of spectra at 15°C, 20°C, and 4°C shows that the imino proton peaks of G18 and T4 disappear when the temperature is increased from 4°C to 15°C, and the imino proton peak of T20 decreases much more than that of T8. These results show that the G18:C5, A19:T4 and T20:A3 base pairs on the 5’-side of the G6* are more destabilized by the *trans*-B[*a*]P-dG adduct than the G16:C7 and A15:T8 base pairs on the 3’-side of G6*. These results are also consistent with the weaker NOEs between C5(NH,H’)—G18(NH1)(m,m’) than those of the 5’-flanking C7(NH,H’)—G16(NH1) base pair (o,o’) (Fig 2B).

#### Non-exchangeable protons

Expanded contour maps of a NOESY spectrum (300 ms mixing time) depicting interactions between the base and the sugar H1’ protons in D_2_O buffer, recorded at 10°C are shown in Fig 3. The base and sugar H1’ assignments were established by cross-checks with other regions of the NOESY plot, and we therefore obtained a complete set of sugar H2’, H2”, H3’ and H4’ proton assignments (Supplementary Information).

The NOE connectivities of base protons and their own and 5’ flanking sugar H1’ protons are traced as blue lines for the modified strand continuously from C1 to C11, except at the T4-C5 and C5-G6* steps (Fig 3A, boxes *b* and *a*, respectively). The NOESY walk for the complementary strand is from G22 to G18 on the 5’-side of the G6*-THF site of the duplex and from G16 to G13 on its 3’-side (Fig 3B). The NOEs are observed for all the resonances. The absence of an NOE between the H8 proton of G6* and the 5’-flanking C5 sugar H1’ proton (Fig 3A, box *a*) is consistent with the absence of the G6* imino proton in the exchangeable proton spectrum (Fig 2) and is also consistent with an abnormal, possibly extrahelical position of the guanine residue of G6*. Furthermore, the NOE between the H1’ proton of G6* and the H6 proton of the 3’-flanking C7 base is weak, and the NOE between H8 of A4 and the H1’ proton of C5 is also very weak (Fig 3A, box b). The observed weak and very weak inter-residue connectivities at the G6*-C7 and T4-C5 steps, respectively, are further evidence of a distorted local structure on the 5’-side of the G6*-THF site, as already noted on the basis of the results derived from Fig 2. The NOESY walk of the THF17-containing strand (Fig 3B) shows a consistently weakened NOE between the H1’ of dG18 and the H8 of dA19. These results further support the greater distortion of the normal Watson-Crick base pairing on the 5’-side of the G6*:THF17 site than on its 3’-side.

We have also analyzed other regions of the NOESY contour plot of the *trans*-B[*a*]P-dG:AB duplex, and a number of unusual chemical shifts are notable. There is a large upfield shift in the base proton H6 (5.96ppm) and the sugar protons H2’ (0.770 ppm) and H2”(1.46 ppm) of C5 (S3 and S4 Tables), suggesting that C5 stacks with the looped-out, displaced G6* base. In addition, the H1’ proton of C7 (4.25ppm) and the sugar H1’ proton (5.031 ppm) of G18 are also shifted upfield, but to a lesser extent. These large proton shifts indicate that C7(H1’) and G18(H1’) stack with the intercalated pyrenyl aromatic ring system, as manifested by the up-field shifts of the G16 and G18 imino protons (Fig 2A).

The base H8 (7.96 ppm), sugar H1’ (6.49 ppm), H2’ (2.64 ppm) and H2” (3.02 ppm) protons of the G6* are shifted downfield, which indicates that the G6* base is, at least in part, displaced from the interior of the double helix and is less well stacked with adjacent bases. The greater distortion of the C5:G18 base pair is also supported by its weakened imino-imino proton cross-peak as shown in Fig 2A.

#### Benzo[*a*]pyrenyl proton connectivities and chemical shifts

**Fig 4:**
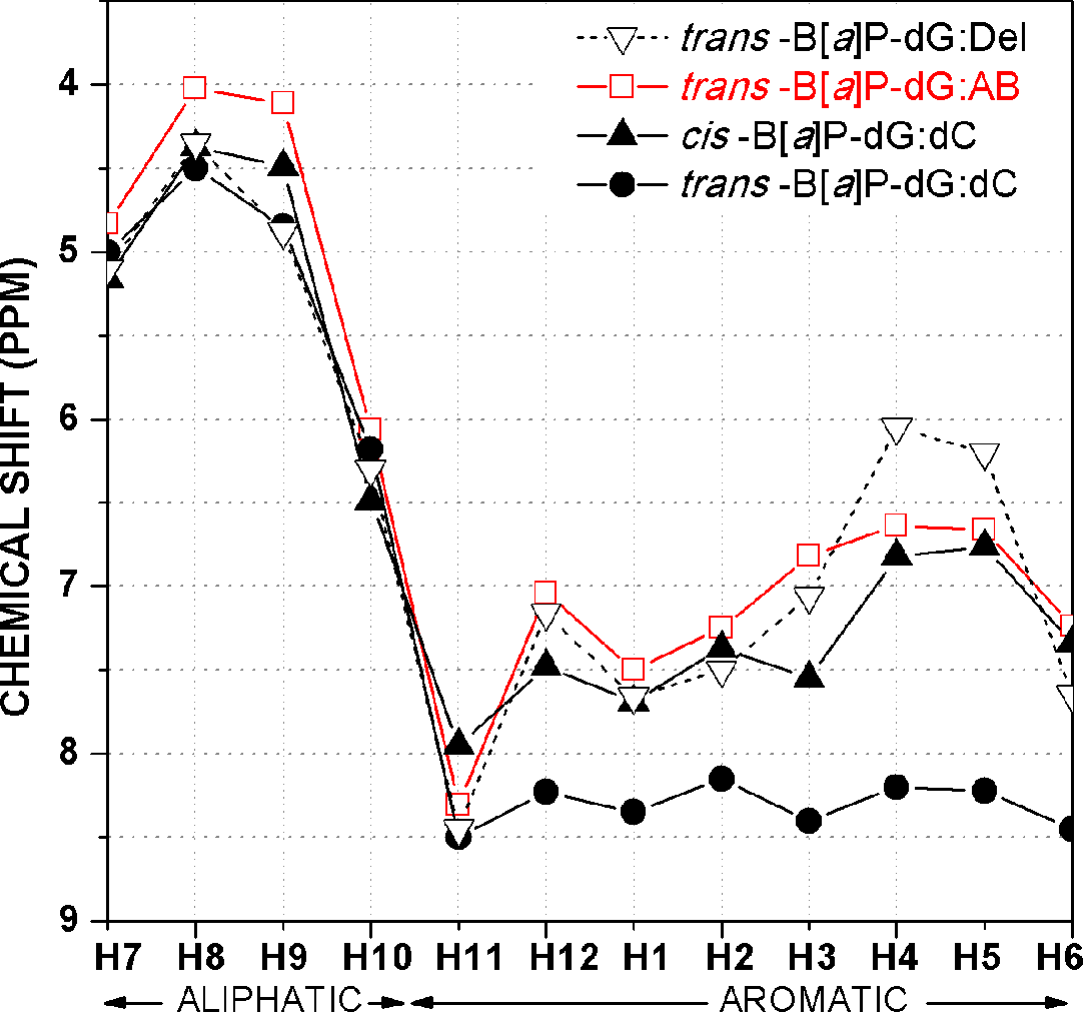
Comparison of chemical shifts of B[*a*]P protons: *trans*-B[*a*]P-dG:AB 11/11-mer duplex (□); *trans*-B[*a*]P-dG:dC duplex in a full, normally base-paired 11/11mer duplex with the aromatic pyrenyl residue in the minor grove [24] (●); stereoisomeric *cis-*B[*a*]P-dG:dC 11/11-mer duplex [22] (▲); *trans*-B[*a*]P-dG:del 11/10-mer duplex [18] (▽).

The non-exchangeable four aliphatic and eight aromatic protons of the B[*a*]P ring system were assigned by analysis of through-space NOESY connectivities and through-bond TOCSY resonances (examples are shown in S2 and S3 Figs). The chemical shifts of the B[*a*]P protons in the *trans*-B[*a*]P-dG:AB duplex are plotted in Fig 4 and compared with those of the stereochemically identical *trans-*B[*a*]P:dC full 11/11mer duplex (with the aromatic B[*a*]P ring system positioned in the minor groove [24], or a *trans*-B[*a*]P-dG 11/10mer deletion duplex (the B[*a*]P aromatic ring system is intercalated [18]). For comparison, we also show the chemical shifts of the stereoisomeric and base-displaced intercalated *cis-*B[*a*]P-dG:dC full 11/11mer duplex [35]. In all previously known cases in which the B[*a*]P aromatic ring system assumes an intercalated conformation [18, 22, 35], the resonances of the H1, H2, H3, H4, H5, H6, and H12 aromatic protons are upfield-shifted (6.0 and 7.5 ppm) (Fig 4). In the case of the *trans*-B[*a*]P-dG:dC full 11/11mer duplex in which the B[*a*]P aromatic ring system is positioned in the minor groove pointing into the 5’-direction of the modified strand, the same protons resonate between 8.0 and 8.5 ppm [24]. This upfield shift phenomenon has been observed in all known cases where the pyrenyl ring adopts a base-displaced intercalative conformation [37]. In the case of the *trans*-B[*a*]P-dG:AB duplex, with the B[*a*]P lesion opposite the THF abasic site, the chemical shifts of all of the aromatic pyrenyl ring protons are upfield shifted, which indicates that the B[*a*]P aromatic residue opposite an abasic site in this duplex is also intercalated.

#### NOEs involving the B[a]P ring system and DNA base and sugar protons

A total of 30 NOEs between the non-exchangeable aromatic B[*a*]P residue protons and nearby exchangeable and non-exchangeable base and sugar protons were identified and assigned (some of these resonances are shown in Fig 3A and S2 Fig) and are summarized in Table 1. Severe cross peak overlaps prevented identification of several other NOEs. However, these NOEs delineate the positioning of the THF and the B[*a*]P ring system. The location of the THF moiety is defined by NOE connectivities between B[*a*]P H2, H3, H4, H5 and H9 protons with the THF H2’ and H2” protons. The positioning of the B[*a*]P ring system in the intercalation pocket is defined by NOEs between B[*a*]P(H2), B[*a*]P(H3), B[*a*]P(H4) and B[*a*]P(H5) protons with DNA protons of G18 and G16 on the THF-containing strand, as well as NOE connectivities between B[*a*]P (H12) and B[*a*]P (H1) with the base and sugar protons of C5. The B[*a*]P benzylic ring position on the major groove side of the DNA duplex is determined by the NOE between the B[*a*]P(H10) and C7(H5).

### NMR distance-restrained *trans*-B[*a*]P-dG structure: Intercalation of the B[*a*]P ring system with displacement of the modified guanine into the major groove opposite THF

**Fig 5:**
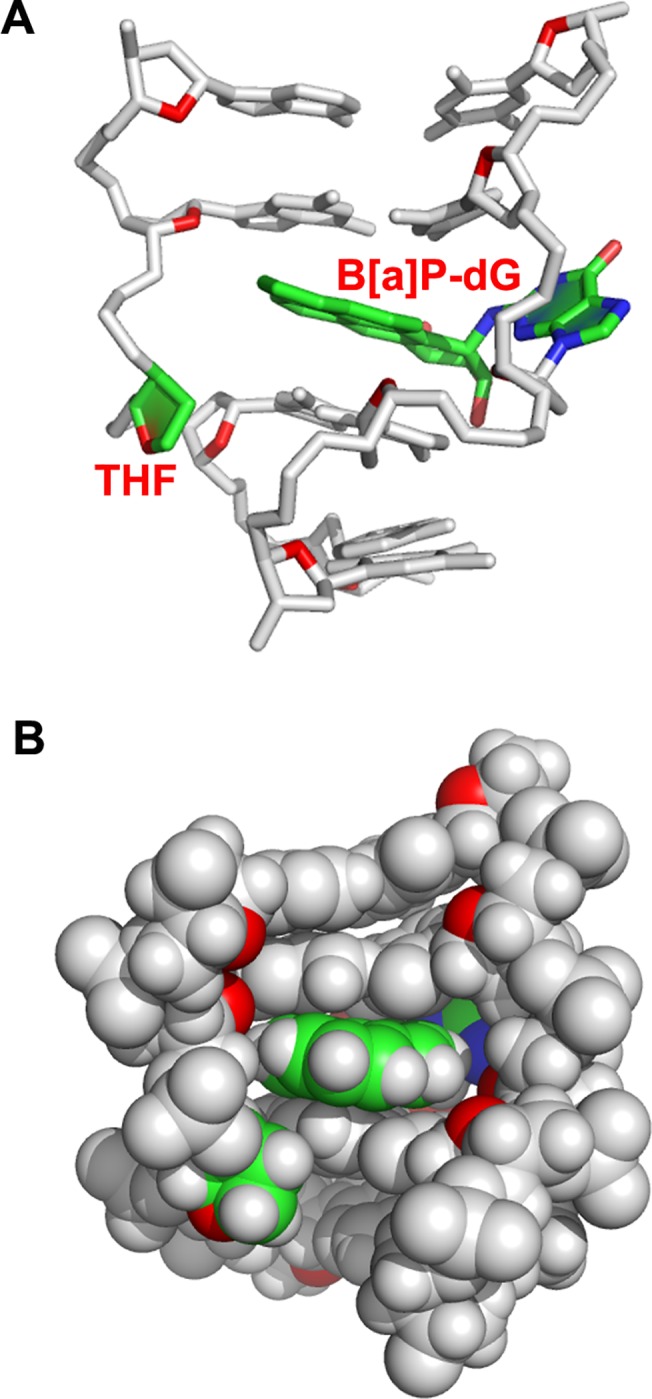
Representative structure of the *trans*-B[*a*]P-dG:AB duplex central 5-mer. The view is into the minor groove. The B[*a*]P rings are intercalated between C5:G18 and C7:G16. The color code is as follows: the *trans*-B[*a*]P-dG adduct opposite the AB site colored by atom; C, green; O, red; N, blue, and the rest of the DNA is in gray. The sugar ring O4’ atom is in red. Hydrogen atoms in the DNA duplex are not displayed for clarity. (A) and (B) are stick and space-filling renderings, respectively.

We utilized MD simulations with distance restraints to obtain structural models consistent with the NOEs and the NMR-derived data. The NMR data indicated that the *trans*-B[*a*]P-dG adopts a base-displaced intercalated conformation, with the B[*a*]P aromatic rings inserted into the helix and the damage guanine displaced; accordingly, we created such an initial model of the *trans*-B[*a*]P-dG adduct opposite THF in a B-DNA duplex, and carried out restrained MD simulations in explicit solvent and with neutralizing sodium ions, as detailed in the Methods section. We then obtained five representative structures from the ensemble (see Methods) derived from the 1 ns restrained MD simulation that represent the NMR data. Table 1 shows the mean achieved distances with standard deviations for these five structures. We note that all achieved inter-proton distances, when their standard deviations are included, are very near the assigned distance bounds. The ensemble of the five structures is shown in S4 Fig and one selected structure is shown in Fig 5. All the analyses described below are the average values for these five structures.

As shown in Fig 5, the B[*a*]P rings intercalate between the C5:G18 and C7:G16 base pairs with the OH-containing benzylic ring on the major groove side and the pyrenyl aromatic rings protruding into the minor groove side. The intercalation is revealed in the NMR data by the upfield shifts of G16 and G18 imino protons (Fig 2) and the B[*a*]P aromatic protons (Fig 4). The modified guanine is extruded into the major groove and is partially stacked with C5, as indicated by the absence of the G6* imino proton resonance (Fig 2), the lack of NOE connectivity at the C5-G6* step, and the upfield shift of the C5 base and sugar protons (S3 Table). The NMR data, specifically the temperature-dependence of the imino proton spectrum (S1 Fig) and weakened inter-residue connectivities (Fig 3), indicate disturbance of Watson-Crick base pairing at the C5:G18 and C7:G16 base pairs, with disturbance greater at C5:G18. Our distance-restrained structures show distorted and fluctuating hydrogen bond angles at these base pairs (S5 Table) that reflect weakened hydrogen bonding [38] compared to normal Watson-Crick base pairs. The other base pairs, except for one base pair at each end, retain normal Watson-Crick hydrogen bonding, a conclusion that is supported by the observed NOEs between imino and amino protons (Fig 2).

The THF moiety is restrained by several NOEs between the B[*a*]P H2, H3, H4, H5 and H9 protons with the THF H2’ and H2” protons. NMR solution structures of THF abasic or AB sites in duplex DNA show that THF/AB sites can be intra or extra-helical or partially extra-helical [39–43]. In our ensemble of five structures (S4 Fig) the O4’ atom is directed outward towards the solvent, because the steric bulk of the intercalated B[*a*]P ring system restrains the THF moiety from adopting an intrahelical conformation (Fig 5 and S4 Fig).

### Conformational features of the *cis*-B[*a*]P-dG adduct in the abasic duplex

It has been shown previously that the minor groove and intercalative B[*a*]P-dG adduct conformations can be distinguished by their UV absorption spectra [44, 45]. This approach makes it possible to evaluate any gross changes in conformation in the case of the (+)-*cis*-B[*a*]P-dG, without the need to synthesize the milligram quantities of site-specifically modified AB duplexes and the subsequent extensive NMR analysis. This information is needed for interpreting the NER activities elicited by this DNA lesion.

The external minor groove and intercalated B[*a*]P-dG adduct conformations can be distinguished by spectroscopic titration experiments in which the 11-mer complementary strands are added in stepwise aliquots to a solution containing a fixed concentration of the oligonucleotide strand containing the B[*a*]P-dG adduct. The absorption spectrum in the 300–360 nm region, corresponding to the absorption spectrum of the B[*a*]P aromatic residue, changes gradually as the full duplexes are formed and these changes become negligible once the 1:1 modified:unmodified strand concentration ratio is exceeded. Two adduct conformations can be distinguished by this method: (1) the formation of intercalative duplexes is accompanied by a hypochromic effect and a final red-shifted absorption maximum at 352–354 nm due to the fully developed duplexes; (2) in the case of the minor groove B[*a*]P-dG adduct, a hyperchromic effect and a final blue-shifted absorption maximum at 346 nm is observed [44, 45].

**Fig 6:**
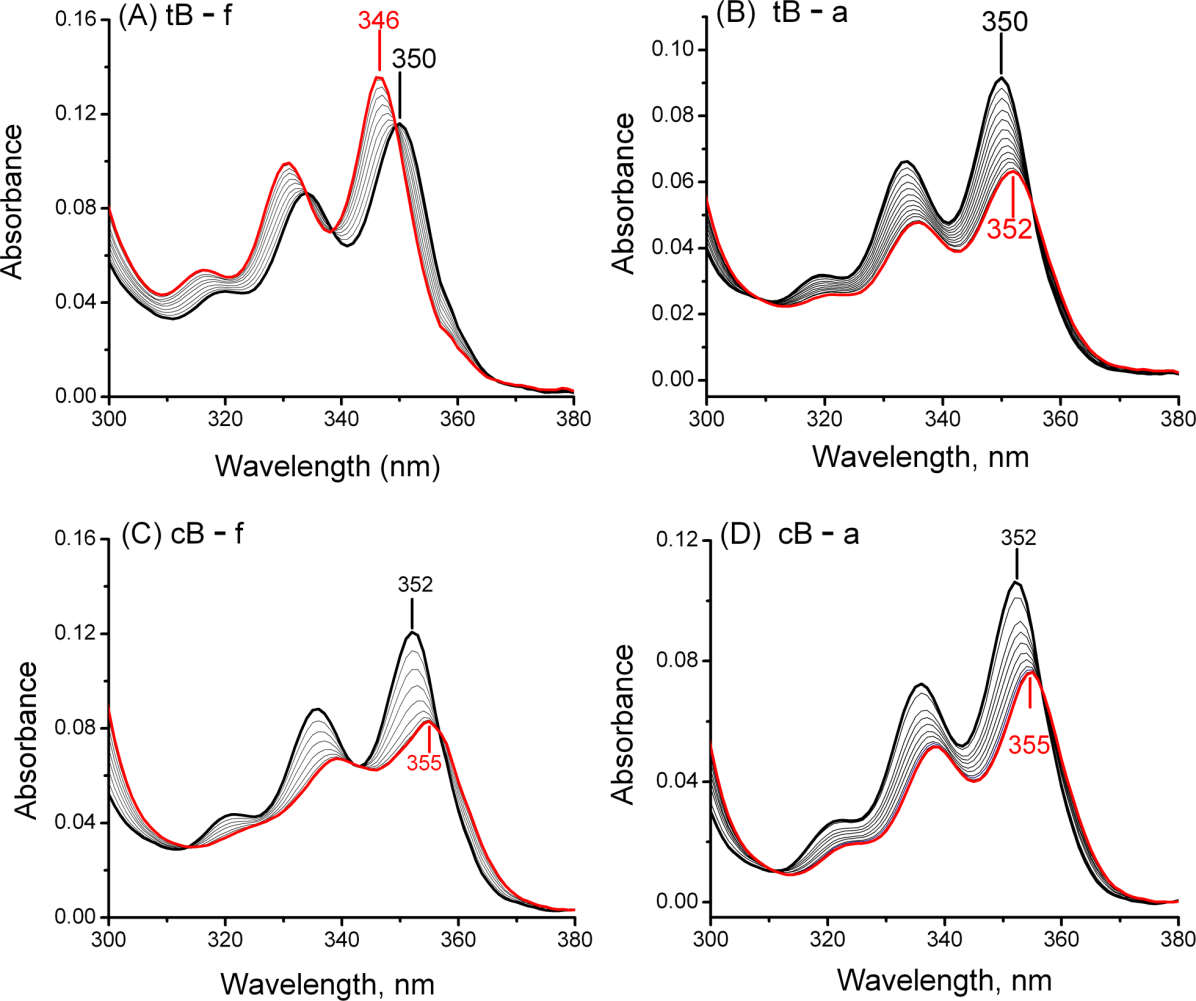
UV absorption spectra differ with intercalated and non-intercalated aromatic ring systems. Titration curves of 11-mer strands containing *trans-*B[*a*]P-dG (A and B) or *cis*-B[*a*]P-dG (C and D) adducts with full complementary 11-mer strands (A and C) or 11-mer strands with abasic THF sites (B and D). The heavy black lines represent the initial absorption spectra of the modified single-stranded oligonucleotides, while the final spectra of the fully titrated duplex end points are denoted by heavy red lines. The intermediate grey lines represent absorption spectra measured at intermediate titration points that correspond to successive additions of aliquots of the complementary strands. Each aliquot contained a 10% concentration relative to that of the fixed concentration of the modified strand. The initial concentrations of the modified strand are ~ 5 μM.

Typical titration experiments are depicted in Fig 6. Denoting the modified *cis*- and *trans*-B[*a*]P-dG strands by cB and tB, and the abasic and fully complementary strands by a and f, respectively, the (cB or tB)/(a or f) ratios ranged from 0.1 to 1.0 in stepwise aliquots, and the absorption spectra were taken after each addition (Fig 6).

As increasing amounts of the fully complementary strand are added to a solution of the *trans*-B[*a*]P-dG modified strand (tB-f), the absorption maximum gradually blue-shifts from 350 nm to 346 nm (Fig 6A) [44, 45]. This result is consistent with the minor groove conformation of this *trans*-B[*a*]P-dG adduct established by NMR methods [24]. If an analogous titration is conducted with the AB-containing strand (tB-a), a small red-shift from 350 nm to 352 nm is observed (Fig 6B); this spectroscopic result is consistent with an intercalated adduct conformation, and is in accord with the NMR results reported in the present study.

In the case of the intercalated *cis*-B[*a*]P-dG strand, titration with the fully complementary strand (cB-f) causes a red-shift in the absorption maximum from 352 to 355 nm in the duplex (Fig 6C). This indicates that the *cis*-B[*a*]P-dG adduct is intercalated, which is consistent with the NMR structure reported by Cosman et al. [35]. Titration of the same cB strand with the AB-containing strand (cB-a), gives rise to a similar red shift with a maximum at 355 nm (Fig 6D). We thus conclude that in the case of the *cis*-B[*a*]P-dG:AB duplex, the aromatic B[*a*]P residue is likewise intercalated with a THF AB site opposite the adduct.

### Melting temperatures

The melting points of the modified 11-mer duplexes are summarized in Table 2. The thermal melting point (T_m_) of the unmodified dG:AB 11/11mer duplex without the B[*a*]P adduct is 26.2±0.5°C; this duplex is thus strongly destabilized relative to the full unmodified 11/11mer duplex with all 11 Watson-Crick base pairs intact (T_m_ = 51±0.5°C, [10]). Thus, the absence of a single base C in the unmodified dG:AB duplex diminishes the T_m_ value by ~ 25°C. The T_m_ of the *trans*-B[*a*]P-dG:AB 11/11mer duplex is 30.5 ±0.5°C, which shows that the *trans*-B[*a*]P-dG adduct tends to stabilize the duplex that also contains an abasic site by ΔT_m_ = T_m_(*mod*)—T_m_(*um*) ~ +4°C, where *um* and *mod* refer to unmodified and B[*a*]P-modified duplexes, respectively. In the case of the stereoismeric (+)-*cis-*B[a]P-dG duplex we obtained a ΔT_m_(AB) = + 17°C value.

**Table 2:** Thermal melting, conformation and repair data for B[*a*]P-dG adducts and sequences investigated.

	*trans*-B[*a*]P-dG			*cis*-B[*a*]P-dG		
Duplex	ΔT_m_ ^*a*^ (°C)	Conformation	NER	ΔT_m_ ^*a*^(°C)	Conformation	NER
Abasic	+4	base-displaced intercalated	resistant	+17	intercalated	resistant
Deletion	+6	base-displaced intercalated	resistant	+19	intercalated	resistant
Full	-10	minor groove	moderate	-11	intercalated	well-repaired

^*a*^ ΔT_m_ = T_m_(*modified*)—T_m_(*unmodified*)

### NER Assays

**Fig 7:**
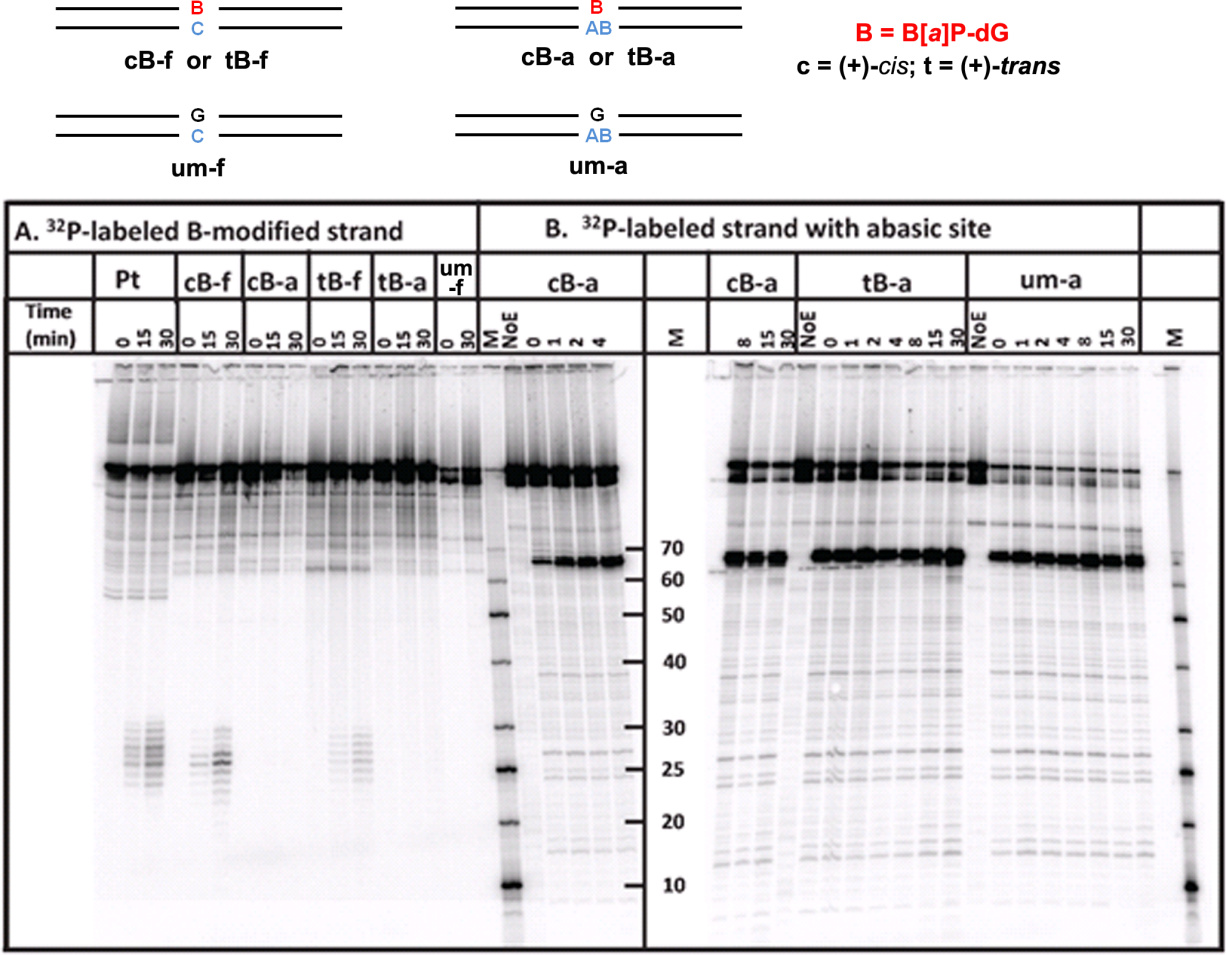
NER assays with HeLa cell extracts. The *trans*-B[*a*]P-dG:AB 135/135mer duplexes were incubated for the time intervals indicated, and the incision products were resolved on 12% denaturing PAGE gels. (A) The modified strand bearing the *trans*-B[*a*]P-dG residue at the 68^th^ nucleotide counted from the 5’-end of the modified strand in this *trans*-B[*a*]P-dG:AB 135/135mer duplex. This duplex was internally ^32^P-labeled between the 60^th^ and 61^st^ nucleotide counted from the 5’-end. The dual incision NER products are 25–30 nucleotides in lengths. **Pt**: A positive control of NER activity: a 135-mer *cis*Pt intrastrand G*CT* cross-linked lesion embedded and centered at the same site as the B[*a*]P-dG adduct in a full and normal 135/135mer duplex, described in detail in [23]; **cB-f**: *cis*-B[*a*]P-dG:dC 135/135mer full and normal duplex (also described in [23]) for comparison of NER activity with the analogous **cB-a** duplex (the *cis*-B[*a*]P-dG:AB abasic 135/135mer duplex); **tB-f** and **tB-a**, analogous duplexes with *trans-*B[*a*]P-dG:dC full and *trans*-B[*a*]P-dG:AB abasic 135/135mer duplexes, respectively; **um-f**: unmodified full 135/135-mer duplex. (B) Only the complementary strand with the THF abasic site at the 68^th^ nucleotide was ^32^P 5’-endlabeled. The incision products ~ 67 nucleotides in length are attributed to base excision repair of the THF abasic site; **cB-a** and **tB-a**, as in (A); **um-a**: unmodified dG:AB 135/135-mer duplex with the THF abasic site at the 68^th^ position counted from the 3’-end.

Results of typical NER assays in human HeLa cell extracts are depicted in Fig 7. In these experiments, the strand carrying the *trans-* or *cis-*B[*a*]P-dG opposite the abasic site in 135-mer duplexes (tB-A and cB-A, respectively), or opposite dC in analogous normal full duplexes (tB-F and cB-F, respectively), were internally ^32^P-labeled (Fig 7, panel A). In these experiments, the incisions of the B[*a*]P-modified strands were assessed. The characteristic ladder of oligonucleotide dual incision products 25–30 nucleotides in length are characteristic of successful NER activity. A G*CT* intrastrand *cis*-Pt adduct (Pt), known to be an excellent substrate of human NER [46] served as a positive control of NER activity [23], while an unmodified 135-mer duplex (um-f) served as a negative control. The *cis*-Pt adduct showed a robust dual incision activity as expected for NER-active cell extracts [23]. The *cis*- and *trans*-B[*a*]P-dG adducts opposite dC in the partner strand in fully complementary duplexes exhibited about the same relative NER activities (cB-f and tB-f, Fig 7, panel A) as observed earlier (*cis/trans* ratio of ~ 5:1) [19, 23]. However, very little NER activity was observed when the same adducts were positioned opposite the abasic THF sites in otherwise identical duplexes (cB-a and tB-a, Fig 7, panel A). Similar results were obtained in six other independent experiments performed at different times with different cell extracts. While HeLa cell extracts prepared from different batches of cells at different times exhibit different activities [11], the differences between dC (~ 3–8% fractions of dual incisions after 30 min incubation intervals) and abasic sites opposite the same B[*a*]P-dG adducts, were reproducible (data not shown). None of the replicates showed any NER activity whatsoever in the case of duplexes with abasic sites opposite the same B[*a*]P-dG adducts that exhibited robust NER activity with a C opposite the same adducts.

In the case of the ^32^P 5’-end-labeled complementary strands, robust incisions at the abasic THF site are evident from the appearance of 67-mer oligonucleotides (cB-a, tB-a, and um-a) Fig 7, panel B), except for lanes NoE which denote identical incubation experiments except that cell extracts were not added to these otherwise identical buffer solutions used in all NER experiments; the NoE samples containing various DNA duplex oligomers were otherwise treated in the same manner as the samples containing the cell extracts. No incisions are observable after performing identical experiments, but leaving out the cell extracts (NoE control experiments, NoE lanes under cB-a, tB-a, and um-a, Fig 7, panel B). The THF abasic site is known to be an excellent substrate of the human APE1 BER enzyme [47–49]. Results published by the O. Lavrik group [47, 48] have shown that natural AB sites opposite *trans-* and *cis-*B[*a*]P-dG adducts are readily incised by APE1 in vitro. Since no incisions are observed in the control experiments indicating that the THF abasic sites are stable under our experimental conditions, the incision activities shown in Fig 7, panel B are attributed to base excision repair activity.

**Fig 8:**
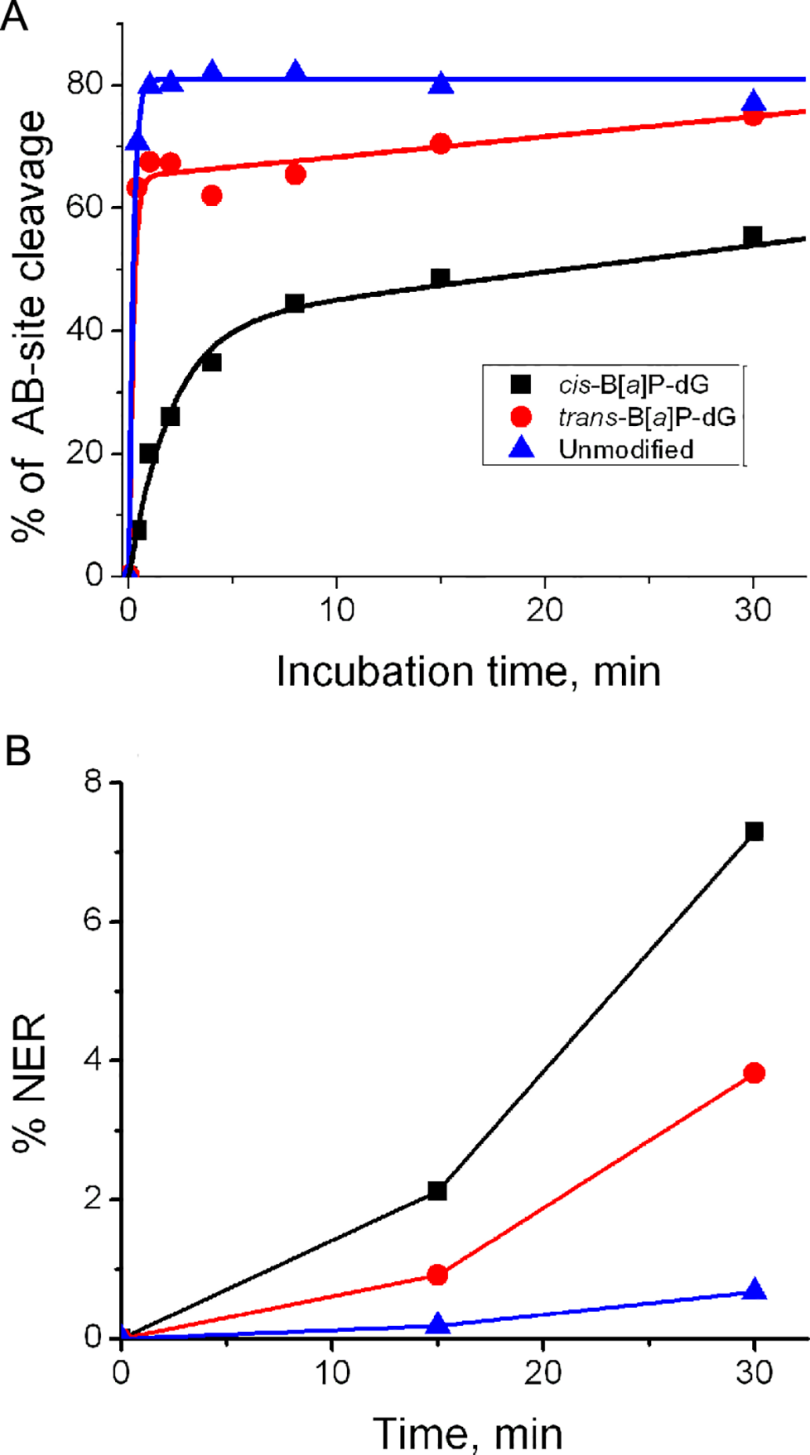
Time course of NER and BER excision cleavage product formation. (A) Time course of cleavage of the DNA strand with the abasic THF site opposite G in the opposite strand either without (unmodified), or with a *trans*- or a *cis-*B[*a*]P-dG adduct (abbreviated by a “B” in this Figure) opposite the AB site in the complementary strand. The solid lines are plots of the standard base excision repair burst equation P = A(1—exp[-*k*t]) + k_ss_ t. The values of A are 81, 65 and 41% for the unmodified, *trans*-B[*a*]P-dG, and *cis*-B[*a*]P-dG adduct-containing complementary strands, with single turnover constants *k* = 5.0, 5.0, and 0.50 min^-1^, and the steady-state constant *k*_*ss*_ = 0, 0.33 and 0.43 min^-1^, respectively. (B) Time course of NER dual incision product formation. All data points were obtained from densitometry analyses of the gel shown in Fig 7.

The individual lanes shown in Fig 7 were analyzed quantitatively by standard densitometry techniques. The fractions of the strands containing the B[*a*]P-dG adducts incised by NER mechanisms (Fig 7A) and strands containing the abasic lesions (Fig 7B), are compared in Fig 8A and 8B, respectively.

#### The resistance to NER of B[*a*]P-dG adducts opposite abasic THF sites is not affected by BER—induced incisions at the abasic site

**Fig 9:**
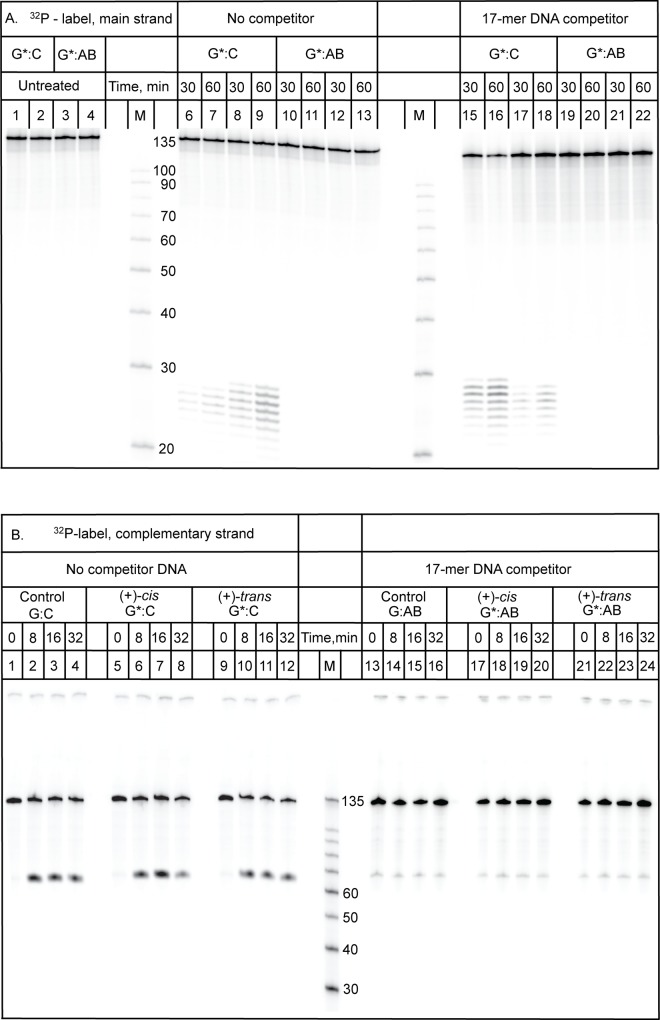
Suppressing the endonuclease-catalyzed BER incisions of the THF abasic site in the complementary strand does not affect the NER dual incisions of the main strand with the B[*a*]P-dG adducts opposite the AB site. Excess amounts (1 μM) of 17-mer competitor DNA duplexes with single THF abasic sites were used to trap the apurinic/apyrimidinic (AP) endonuclease in HeLa cell extracts, thus suppressing the incisions of the complementary strand in the 135-mer duplexes. The sequence context of the 17-mer competitor duplex was (5’-CCACCAACGCTACCACC)•(5’-GGTGGTAGCGTTGGTGG). Incubation in HeLa cell extracts of 135-mer duplexes either with unmodified G:C, modified G*:C or G*:AB sites (G* = B[*a*]P-dG). Panel A: NER activity observed with 135-mer duplexes containing ^32^P-endlabeled main strands with either G* or unmodified G at the same site. Lanes 5 and 14: size markers (M). Panel B: BER activity at the AB sites observed with the same 135-mer duplexes but with ^32^P-endlabeled complementary strands containing either the THF abasic site or unmodified C opposite G*. Lanes 1,6,7,17 and 18: (+)-*trans*-G* opposite C (tB-f in Fig 7); Lanes 2,8,9,15, and 16 (NER activity): (+)-*cis*-G* opposite C (cB-f); Lanes 3,10,11,21, and 22: (+)-*trans*-G* opposite AB (tB-a); Lanes 4,12,13,19, and 20: (+)-*cis*-G* opposite AB (tB-a).

The BER incision products accumulate rapidly (within several minutes), while NER dual incision products accumulate more slowly as a function of time (Fig 8). We therefore investigated whether the BER activity that occurs in parallel with the NER dual incisions (Figs 7 and 8) has any impact on the efficiency of these dual incisions. In principle, the endonuclease APE I-induced incisions could produce a nick in the complementary strand that could alter the conformation of the B[*a*]P-dG adduct opposite the intact THF abasic site deduced from the NMR data. In order to verify that the resistance to NER is indeed associated with an intact abasic site opposite B[*a*]P-dG adducts (G*:AB), we suppressed the BER incisions at the THF abasic site by employing selective competition experiments. We used an excess (1 μM) of a 17-mer oligonucleotide strand with a single THF abasic site (5’-CCACCAACGCTACCACC) annealed with its fully complementary strand as a competitor for BER proteins in HeLa cell extract NER assays. The NER (Fig 7A) and BER (Fig 7B) incisions induced in 135-mer G*:C and G*:AB duplexes were then compared in the absence and presence of 1 μM 17-mer competitor duplexes. In Fig 9A, all 135-mer duplexes contained the ^32^P-labels at the 5’-end of the main strand containing the adduct G*, while in Fig 9B the radioactive label was positioned at the 5’-end of the complementary strand containing the AB site. In Fig 9A, lanes 1–4 depict control experiments showing the bands due to G*:C or G*:AB 135-mer duplexes that were not treated in HeLa cell extracts. Lanes 6–14 are also control experiments without 17-mer competitor duplexes showing the normal NER bands in the case of the same G*:C duplexes (lanes 6–10), and the lack of NER bands in the case of the same G*:AB duplexes (lanes 11–14); these results are fully consistent with those shown in Fig 7. The effects of 17-mer duplexes with a single AB site are shown in lanes 15–18 for G*:C 135-mer duplexes; the 17-mer competitor duplexes do not significantly affect the formation of NER dual incisions of the main strand, as expected. Furthermore, as in Fig 7, there are no NER bands in the case of G*:AB duplexes in lanes 19–22. These results demonstrate that the presence of an excess of BER protein-competitor duplexes does not affect the resistance of the 135-mer G*:AB duplexes to NER.

By contrast, base excision repair of the ^32^P-labeled complementary strand (Fig 9B) is strongly affected by the presence of the same concentration of competitor strands as in Fig 9A. Normal incisions at the AB sites are observed in 135-mer unmodified G:C duplexes (lanes 1–4), in G*:C duplexes (lanes 5–12), and G*:AB duplexes with either (+)-*cis-* or (+)-*trans*-B[*a*]P-dG adducts (lanes 9–12). However, in the presence of 1 μM 17-mer AB-competitor duplexes, the incision of AB sites in the same set of duplexes as in panel A is reduced by 80–85%, and is independent of the absence or presence of the B[*a*]P-dG adduct, or its stereochemical characteristics. The lack of NER signal in the case of G*:AB sites with 80–85% of the AB-containing complementary strands intact (Fig 9B, lanes 17–24), supports the basic conclusion of this work that the (+)-*cis*- and (+)-*trans*-B[*a*]P-dG adducts are indeed resistant to NER when positioned opposite *intact* abasic sites in the complementary strand.

## Discussion

In order to evaluate the effects of removal of the partner base C, while leaving the phosphodiester backbone intact, we have characterized the NMR solution structure of the *trans*-B[*a*]P-dG adduct opposite a tetrahydrofuran THF abasic site, a stable analogue of a natural abasic site (Fig 1). Naturally formed AB sites that are produced as a result of deglycosylation are unstable, because of the residual C1’-OH group, while in THF the—OH group is replaced by an H-atom. We have also investigated the NER susceptibilities of this same *trans*-B[*a*]P-dG adduct, as well as the stereoisomeric *cis*-adduct opposite the THF sites in HeLa cell extracts. A goal was to determine how the NER susceptibility of these lesions was impacted by the absence of the partner base, and to relate the results to the structural features of these bulky DNA lesions.

### Incision of the THF abasic site in the complementary strand by BER proteins

Abasic sites are in themselves very common, genotoxic lesions. They are readily removed by a dedicated enzyme, APE1, a well characterized mammalian base excision repair (BER) enzyme that hydrolyzes AB sites by cleaving the phosphodiester bond on their 5’- and 3’-sides [50, 51]. APE1 also excises *in vitro* the synthetic THF sites [49] that are widely utilized to study the properties of the natural, unstable AB sites; these are comprised of an equilibrium mixture of ring-opened and closed tautomers, including α and β anomers of deoxyribofuranose. It was found earlier that 10*S*-*trans* and 10*R*-*cis*-B[*a*]P-dG adducts positioned opposite AB sites affect the processing of the latter by BER proteins in a manner that depended on the stereochemistry of these bulky adducts [47, 48].

The presence of the B[*a*]P-dG lesion does not, however, prevent the repair of the THF abasic site by the BER mechanism as shown in the present work and earlier [48]. The mammalian APE1 endonuclease is dedicated to excision of AB sites through incisions on the 5’ and 3’-sides of the AB site, to leave a 3’-OH and a 5’-phosphate group with a one nucleotide gap for subsequent repair by Pol β and ligase. It has recently been shown that the gap remaining following processing of an AB site by APE1 opposite the *trans*-B[*a*]P-dG and *cis*-B[*a*]P-dG lesions can be filled in by Pol β [48].

The THF abasic site is rapidly repaired in the HeLa cell extracts by BER mechanisms [49], as also shown previously using purified APE1 proteins and natural AB sites opposite the same B[*a*]P-dG adducts [47–49]. The kinetics of incision of the THF abasic site-containing strand are clearly biphasic and exhibit a rapid burst phase followed by slower linear steady-state kinetics (Fig 8A). While the kinetics of the incision of abasic sites were not further investigated, the results indicate that the single turnover kinetics are somewhat slower in the case of the *cis-* than the *trans*-B[*a*]P-dG adduct opposite the THF abasic site (Fig 8B).

**Fig 10:**
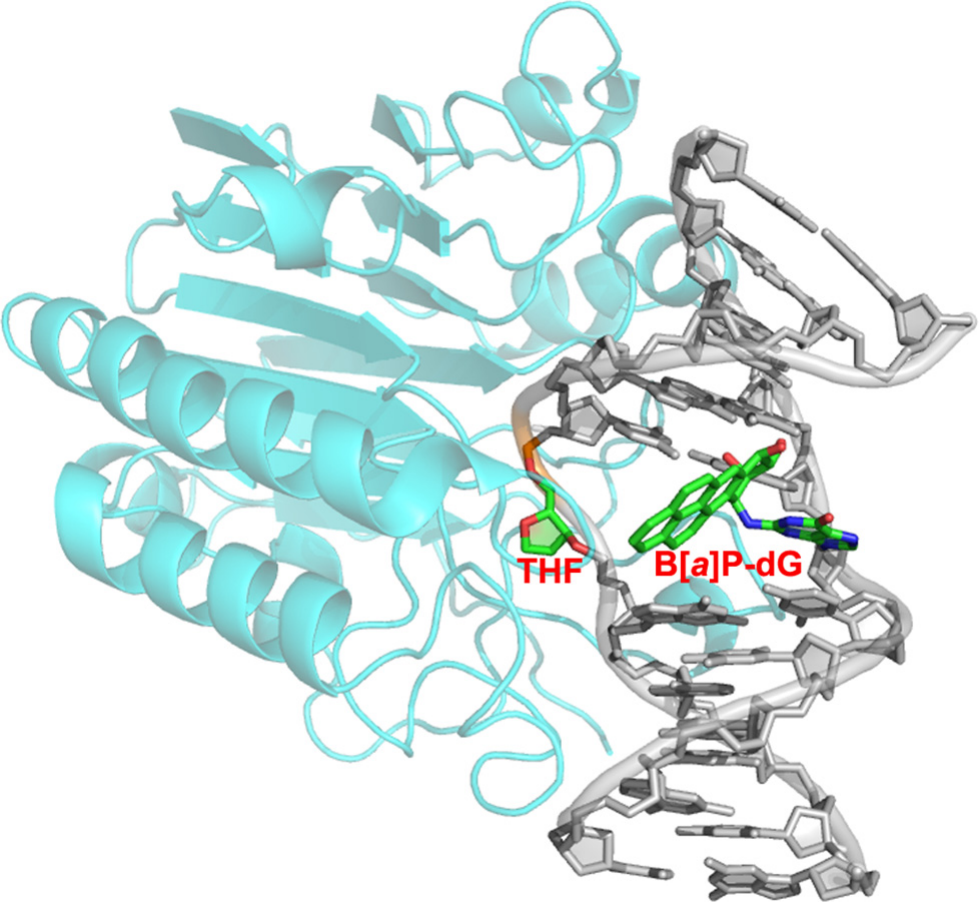
*Trans-*B[*a*]P-dG adduct modeled opposite an AB site in APE1 complexed with duplex DNA [52]. The model shows that the *trans*-B[*a*]P-dG does not disrupt the APE1-DNA-AB structure when placed on the major groove side of the DNA duplex.

A crystal structure of human APE1 bound to a DNA duplex containing a THF site shows the THF residue flipped into the APE1 endonuclease, which bends the DNA helix to engulf the THF-DNA strand via a positively charged surface [52] for specific lesion recognition. To gain further insights into the structural reasons that the B[*a*]P-dG lesion does not prevent recognition of the THF by APE1 [48], we carried out molecular modeling to elucidate how the lesion can be accommodated in the crystal structure of APE1 containing THF in the active site (PDB [53] ID: 1DE8) [52]. Details of the modeling are given in S1 Methods. We hypothesized that the DNA must essentially preserve its structure in the enzyme because of the rigid and pre-formed nature of the enzyme/DNA binding site, which does not change between the apo and the DNA-bound structure [52]. We found that both the present base-displaced intercalated NMR solution structure and the minor groove orientation of the B[*a*]P ring system in the full duplex [24] cause severe collisions between the bulky B[*a*]P ring system and the DNA and the protein. However, a structure which places the B[*a*]P rings in the major groove was feasible. In this location the B[*a*]P-dG does not interfere with the binding of the APE1 protein and its recognition of the THF lesion (Fig 10), consistent with the BER susceptibility of the THF even with the B[*a*]P-dG lesion present on the strand opposite the THF site. Another interesting point is that the binding of the APE1 to the THF-containing strand might compete with the binding of the XPC, which binds to the usually undamaged strand that is the partner to the NER susceptible lesion [21]; this effect might contribute to the NER resistance of the *trans*-B[*a*]P-dG lesion in the THF-containing duplex.

### Excision of the B[*a*]P-dG adducts by the NER apparatus

The minor groove *trans*-B[*a*]P-dG lesion is generally removed by the NER system in HeLa cell extracts with ~ 5 times lower efficiencies than the stereoisomeric *cis*-B[*a*]P-dG adduct in the same sequence context in fully complementary 135/135mer duplexes [19, 23], with partner dC bases opposite the modified guanines, as used in this study. Our NMR results reveal that the removal of the partner base in the case of the *trans*-B[*a*]P-dG adduct changes the lesion conformation from an external minor groove to a base-displaced intercalated one. However, no significant changes in conformations are observable in the case of the *cis*-B[*a*]P-dG adduct which is intercalated in the full duplexes [35], as well as in the abasic duplexes (Fig 6). Neither the *cis-* nor the *trans*-B[*a*]P-dG adduct opposite intact abasic THF sites are incised by the human NER system under our experimental conditions (Figs 7–9), although the fully complementary control duplexes containing the same stereoisomeric B[*a*]P-dG adducts are incised normally as also shown previously [19, 23].

The structures and thermal melting data provide insights into the NER resistance for the *trans*- and *cis*-B[*a*]P-dG:AB duplexes. The B[*a*]P ring system is in the B-DNA minor groove in the normal *trans*-B[*a*]P-dG duplex opposite dC with all Watson-Crick pairs intact [24]. However, in the abasic THF-containing *trans*-B[*a*]P-dG duplex, the NMR data show that the B[*a*]P rings are intercalated to stack with adjacent base pairs C5:G18 and C7:G16, whose Watson-Crick pairs are perturbed but not ruptured. The intercalation of the B[*a*]P ring system is from the major groove side where the displaced guanine moiety resides. The local stabilization provided by the stacking interactions in the *trans*-B[*a*]P-dG:AB duplex compared to the dG:AB duplex that lacks the B[*a*]P lesion is reflected in the difference in the melting points of the duplexes ΔT_m_(AB) = T_m B[a]P-dG:AB—_T_m dG:AB_ = + 4°C in the case of the (+)-*trans*-B[*a*]P-dG:AB duplex 11-mer. In the case of the stereoismeric *cis-*B[*a*]P-dG duplex that is also intercalated according to spectroscopic data, ΔT_m_(AB) = + 17°C. These results, indicate that the bulky polycyclic aromatic residues stabilize the THF AB duplexes by stacking interactions; this is analogous to the effects exerted by the same adducts on the stabilities of deletion duplexes that lack the entire nucleotide in the complementary strand opposite the B[*a*]P-dG adduct, and also adopt base-displaced intercalated conformations [18, 22]: ΔT_m_(Del) = T_m B[a]P-dG:Del—_T_m dG:Del_ = +19°C in the case of the (+)-*cis*-B[*a*]P-dG [12] or ΔT_m_(Del) = +6°C for the (+)-*trans* adduct [37]. In sharp contrast, the same stereoisomeric B[*a*]P-dG adducts in fully complementary 11-mer duplexes destabilize the double-stranded DNA by ΔT_m_ (Full) = -11°C ((+)-*cis*), and = -10°C ((+)-*trans*) [37]. We conclude that the removal of the cytosine base opposite the two B[*a*]P-dG adducts has a similar impact as removing the full nucleotide on their adduct conformations, thermodynamic stabilities of the DNA duplexes, and abrogation of NER activities as summarized in Table 2. Differences in B[*a*]P DNA stacking properties due to stereoisomeric effects are likely responsible for the different impacts on the ΔT_m_ values of the base-displaced intercalated *cis*-and *trans*-B[*a*]P-dG adducts [18, 22]

### NER resistance is correlated with local thermodynamic stabilization

Based on the crystal structure of the yeast ortholog of the human XPC-HR23B NER recognition protein containing a CPD lesion [21] we hypothesize that the NER resistance of the *trans*- and *cis*-B[*a*]-dG lesions opposite an AB site stems in part from the *local* stabilization provided by the intercalated B[*a*]P ring system, which would impede insertion of the XPC BHD3 β hairpin. Furthermore, the absence of the partner base would weaken binding of the protein to the damaged DNA because the binding affinity of XPC depends on a competition between DNA opening and the time spent by the XPC protein at the lesion site [21, 54]. Since two flipped out partner bases stabilize the open XPC—DNA lesion structure, the missing single complementary strand opposite the lesion in G*:AB duplexes may significantly reduce the residence time of XPC and thus the subsequent recruitment of TFIIH that ultimately leads to the formation of the dual incision products. Similar explanations are plausible for the NER resistance of the intercalated *trans*- and *cis*-B[*a*]P-dG lesions in the deletion duplexes [12, 19]. Additionally, it has been shown that the topology of the intercalated lesion also plays an important role in determining whether any NER is observed in the deletion duplexes, as discussed previously for the smaller food mutagen-derived three-ringed 2-amino-1-methyl-6-phenylimidazo[4,5-b]pyridine (C8-dG-PhIP) adduct [12].

## Conclusion

The structural properties of DNA lesions that foster or inhibit NER remain of fundamental interest from a mechanistic perspective, and defining them has important implications for elucidating the characteristics that allow lesions to persist and cause harmful mutations. We have found that the missing partner base in the abasic site on the complementary strand opposite the *trans*- and *cis*-B[*a*]P-dG lesions affects two important characteristics of the local adduct binding sites that impact the downstream NER dual incision events. The intercalation of the bulky aromatic B[*a*]P residues locally stabilizes the DNA duplex by stacking interactions with adjacent base pairs; we hypothesize that this local stabilization hinders the strand separation near the lesion that is required for the insertion of the β-hairpin and thus the binding of the XPC-RAD23B NER recognition factor to the damaged DNA site. In addition, the absence of one of the complementary strand bases opposite the DNA adduct, that would normally be flipped into the XPC protein, weakens the interactions of the damaged DNA site with the protein [21]. Since the β-hairpin insertion and base flipping may or may not be coupled events depending on the nature of the lesion, the sequence of events must be investigated experimentally and computationally on a lesion-by-lesion basis.

## Supporting Information

S1 Fig Series of 1D spectra recorded in H_2_O buffer at different temperatures. (DOCX)

S2 Fig Expanded contour plot of a NOESY spectrum (300 ms mixing time) showing the NOE connectivities of abasic 17 and G18 protons. (DOCX)

S3 Fig Comparison of expanded contour plot of TOCSY and NOSEY spectra. (DOCX)

S4 Fig Superpositioned five best representative structures of the 10*S*-B[*a*]P-dG:AB duplex from the restrained MD simulation. (DOCX)

S1 Methods Molecular modeling and molecular dynamics protocol. (PDF)

S1 Table Force field parameters for the THF site. (DOCX)

S2 Table Force field parameters for the *trans*-B[*a*]P-dG adduct. (DOCX)

S3 Table Chemical Shifts of the *trans*-B[*a*]P-dG:AB 11mer Duplex in D2O. (DOCX)

S4 Table Nucleic acid chemical shifts of the 11 mer unmodified duplex. (DOCX)

S5 Table Average values of hydrogen bond angles. (DOCX)
