# Correction: Resistance to Nucleotide Excision Repair of Bulky Guanine Adducts Opposite Abasic Sites in DNA Duplexes and Relationships between Structure and Function

**DOI:** 10.1371/journal.pone.0142068

**Published:** 2015-10-29

**Authors:** Zhi Liu, Shuang Ding, Konstantin Kropachev, Lei Jia, Shantu Amin, Suse Broyde, Nicholas E. Geacintov

The fourth author’s name is spelled incorrectly. The correct name is: Lei Jia. The correct citation is: Liu Z, Ding S, Kropachev K, Jia L, Amin S, Broyde S, et al. (2015) Resistance to Nucleotide Excision Repair of Bulky Guanine Adducts Opposite Abasic Sites in DNA Duplexes and Relationships between Structure and Function. PLoS ONE 10(9): e0137124. doi:10.1371/journal.pone.0137124
