# Supplementary material for: Resistance to Nucleotide Excision Repair of Bulky Guanine Adducts Opposite Abasic Sites in DNA Duplexes and Relationships between Structure and Function
Source: PLoS One. 2015 Sep 4;10(9):e0137124. doi: 10.1371/journal.pone.0137124 (PMC4560436; doi:10.1371/journal.pone.0137124)
Supplement: S1 Fig — (DOCX) [file pone.0137124.s001.docx]

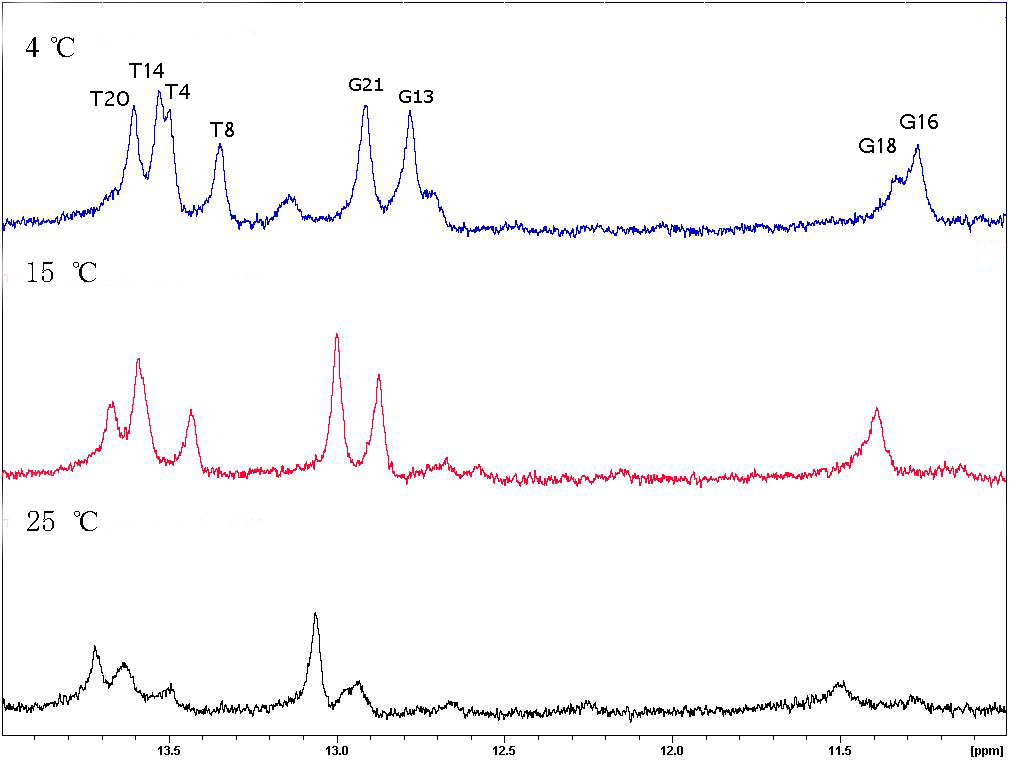


Figure S1. Series of 1D spectra recorded in H_2_O buffer solution at 500MHz at different temperatures. Imino proton assignments are shown in the spectrum recorded at 10 ºC. Note the different behavior of the G18, G16, T4 and T20 imino protons as a function of temperature.
