# Supplementary material for: Resistance to Nucleotide Excision Repair of Bulky Guanine Adducts Opposite Abasic Sites in DNA Duplexes and Relationships between Structure and Function
Source: PLoS One. 2015 Sep 4;10(9):e0137124. doi: 10.1371/journal.pone.0137124 (PMC4560436; doi:10.1371/journal.pone.0137124)
Supplement: S2 Fig — (DOCX) [file pone.0137124.s002.docx]

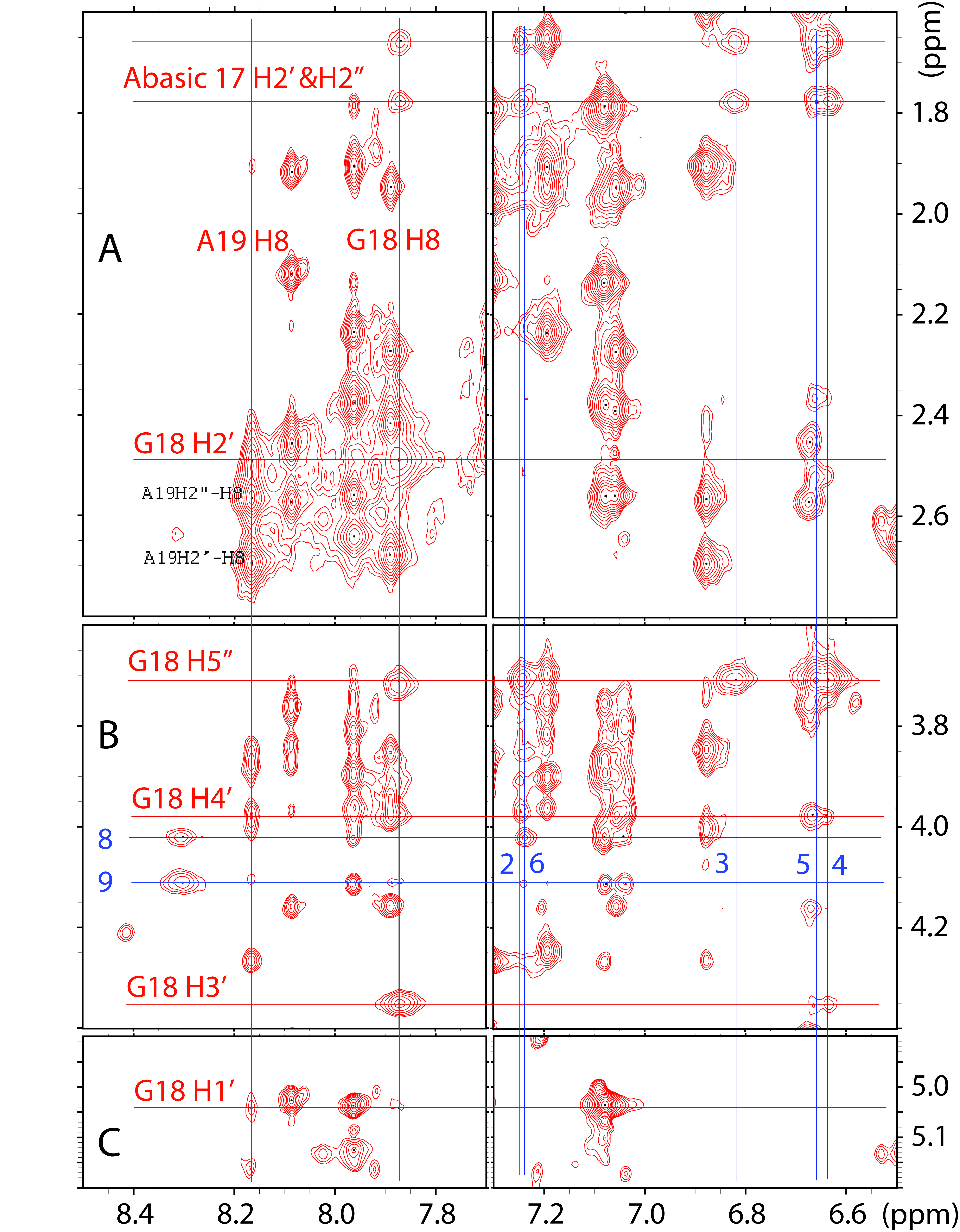


Figure S2. Expanded contour plot of a NOESY spectrum (300 ms mixing time) showing the NOE connectivities of abasic 17 and G18 protons at A: H2’, H2” region; B: H3’, H4’ and H5’ region; C: H1’ region. The NOE connectivities to lesion protons are also marked in contour plot.
