# Supplementary material for: Resistance to Nucleotide Excision Repair of Bulky Guanine Adducts Opposite Abasic Sites in DNA Duplexes and Relationships between Structure and Function
Source: PLoS One. 2015 Sep 4;10(9):e0137124. doi: 10.1371/journal.pone.0137124 (PMC4560436; doi:10.1371/journal.pone.0137124)
Supplement: S3 Fig — (DOCX) [file pone.0137124.s003.docx]

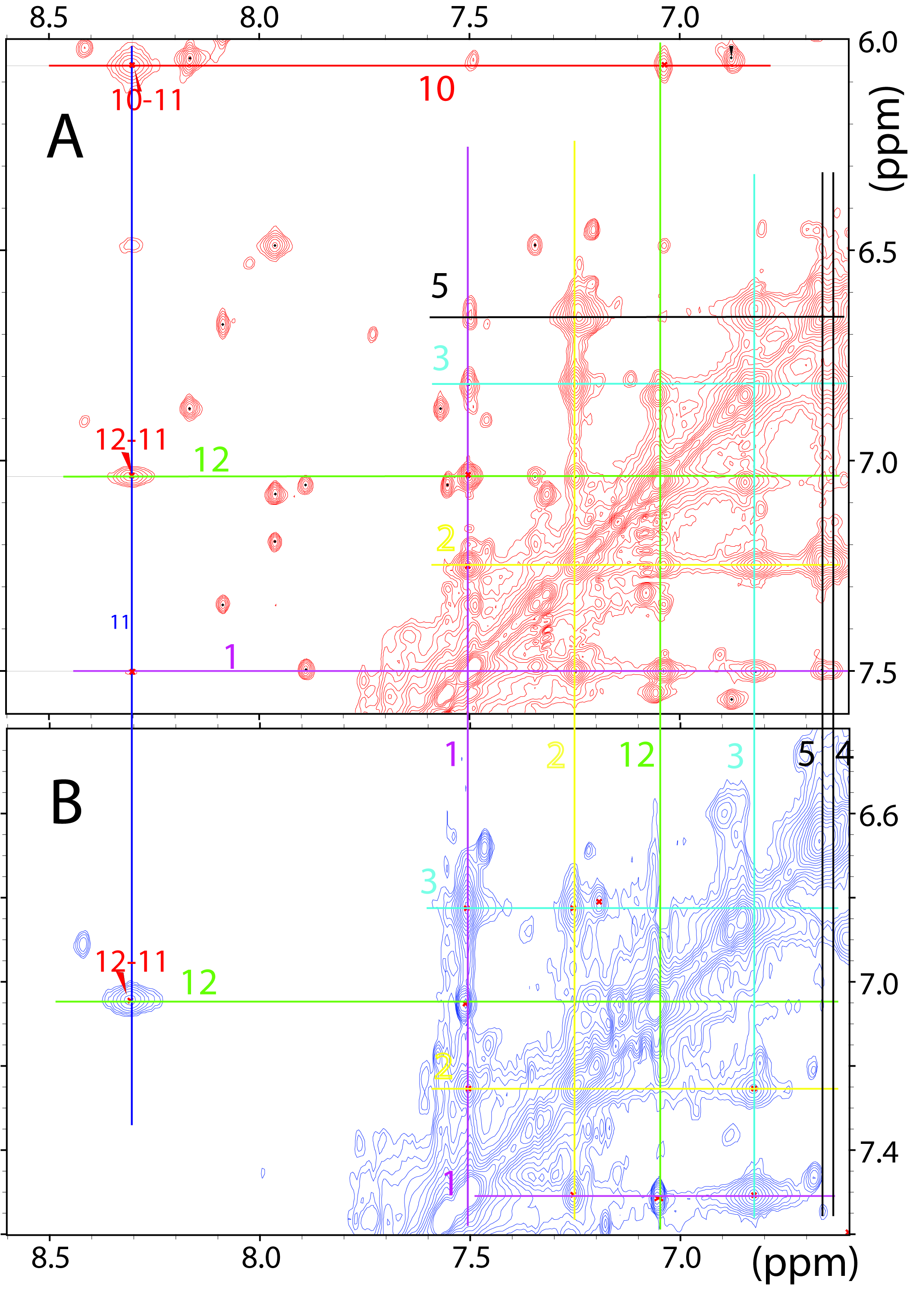


Figure S3. Comparison of expanded contour plot of TOCSY (B) and NOSEY (A) spectra of the 10*S*-B[*a*]P-dG:AB duplex in D_2_O aqueous buffer solution measured in a 500MHz spectrometer.
