# Supplementary material for: Resistance to Nucleotide Excision Repair of Bulky Guanine Adducts Opposite Abasic Sites in DNA Duplexes and Relationships between Structure and Function
Source: PLoS One. 2015 Sep 4;10(9):e0137124. doi: 10.1371/journal.pone.0137124 (PMC4560436; doi:10.1371/journal.pone.0137124)
Supplement: S4 Fig — (DOCX) [file pone.0137124.s004.docx]

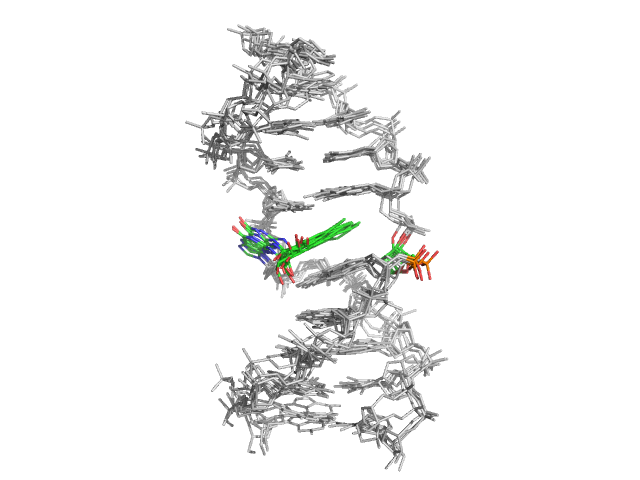


Figure S4. Superpositioned five best representative structures of the 10*S*-B[*a*]P-dG:AB duplex from the restrained MD simulation; the view is into the major groove. The 10*S*-B[*a*]P-dG adduct and THF site are colored by atom: carbon, green; nitrogen, blue; oxygen, red; phosphorus, magenta, and the rest of the DNA is in gray. Hydrogen atoms in the DNA duplexes are not displayed for clarity.
