# Supplementary material for: Resistance to Nucleotide Excision Repair of Bulky Guanine Adducts Opposite Abasic Sites in DNA Duplexes and Relationships between Structure and Function
Source: PLoS One. 2015 Sep 4;10(9):e0137124. doi: 10.1371/journal.pone.0137124 (PMC4560436; doi:10.1371/journal.pone.0137124)
Supplement: S1 Methods — (PDF) [file pone.0137124.s005.pdf]

Methods

Molecular Modeling

The NMR data indicated that the *trans*-B[*a*]P-dG adduct opposite a THF AB site adopts the base-displaced intercalated conformation in the 11-mer duplex with the THF moiety opposite the lesion. In contrast, this same adduct is positioned in the minor groove with a normal partner dC opposite the adduct [1]. To create an initial B-DNA model for this adduct that is base-displaced intercalated, we started with the NMR solution structure of the base-displaced intercalated 10*S*-(–)-*cis*-B[*a*]P-dG adduct in the same 11-mer duplex sequence [2]; we interchanged the H and OH groups on C7, C8 and C9 to generate the base-displaced intercalated 10*S*-(+)-*trans*-B[*a*]P-dG stereoisomer. The THF moiety was generated by cutting the glycosidic bond to the C17 base that was present in the 10*S*-(–)-*cis*-B[*a*]P-dG NMR solution structure, and replacing it with one hydrogen atom. This initial model was subjected to 15 ns unrestrained MD simulation for equilibration.

Molecular Dynamics Protocol

MD simulations were carried out with the SANDER module from the AMBER 9.0 suite [3]. The cutoff value of the nonbonded interactions was set at 9.0 Å. The SHAKE algorithm [4] was employed to constrain bonds involving hydrogen with a tolerance of 10^{-6}Å. A 2 fs time step was used in the dynamics simulations, and the translational motion of the center of mass was removed every 1 ps [5]. The particle mesh Ewald (PME) method [6,7] was applied to treat long range electrostatic interactions. To neutralize the solute, 20 Na^+ counterions were added to the modified duplex, using the LEAP module. The system was solvated by adding a rectangular box of TIP3P water [8] 10 Å from the boundary of the DNA in each direction. To relax the solvent,

the solute DNA was held fixed by 25 kcal/mol restraints, and the system was minimized with 1000 steps of steepest descent minimization. This was followed by a 25ps MD simulation with the same constraints on the DNA under constant volume. At this stage, the temperature was raised from 10 K to 300 K during the first 20 ps, using the Berendsen coupling algorithm [9] with a coupling parameter of 1.0 ps. Then, 5 rounds of minimization with 600 steps of steepest descent minimization in each round were performed to uniformly decrease the restraints on the DNA from 25 kcal/mol to 0 kcal/mol [10]. Next, a 60 ps MD simulation was applied to equilibrate the system under constant volume, with the temperature raised uniformly from 10 K to 300 K in the first 40 ps. Finally, an MD simulation of 15 ns was carried out under constant pressure for the production.

The partial charges and other force field parameters for the *trans*-B[*a*]P-dG adduct were taken from [11]. Partial charges for the THF moiety were computed utilizing quantum mechanical Hartree Fock calculations with the 6-31G* basis set employing the Gaussian 03 package [12]. The charges were then fitted to each atomic center with the RESP algorithm [13]. Other force field parameters for the THF were assigned to be consistent with the rest of the AMBER force field. S1 and S2 Tables give the force field parameters for the THF and the *trans*-B[*a*]P-dG adduct.

Modeling the trans-B[a]P-dG Lesion Opposite An AB Site in APE1

A crystal structure of human APE1 bound to a DNA duplex containing a THF site shows the THF residue flipped into the APE1 endonuclease, which bends the DNA helix to engulf the THF-DNA strand via a positively charged surface [14] for specific lesion recognition. To gain

further insights into the structural reasons that the B[*a*]P-dG lesion does not prevent recognition of the THF by APE1 [15], we attempted to replace the guanine base opposite the THF site in the APE1/THF crystal structure (PDB [16] ID: 1DE8) [14] with the B[*a*]P-G in our NMR solution structure (Fig. 10) using the INSIGHTII program (Accelrys Software, Inc.). We hypothesize that the DNA must essentially preserve its structure in the enzyme because of the rigid and pre-formed nature of the enzyme/DNA binding site, which does not change between the apo and the DNA-bound structure [14]. However, the present NMR solution structure is not feasible in the enzyme without rearranging the DNA, as the intercalated B[*a*]P ring system collides severely with the kinked DNA duplex and protein. We similarly modeled the minor groove orientation of the B[*a*]P-dG, seen in the NMR solution structure of the full duplex [1], into the crystal structure; in this case as well the B[*a*]P rings collide severely with the DNA and protein. However, a structure which places the B[*a*]P rings in the major groove was feasible (only the glycosidic torsion of the B[*a*]P modified guanine was changed from -75º to -88º). In this location the B[*a*]P-dG does not interfere with the binding of the APE1 protein and its recognition of the THF lesion (Fig. 10), consistent with the BER susceptibility of the THF even with the B[*a*]P-dG lesion present on the strand opposite the THF site.

References

1. Cosman M, de los Santos C, Fiala R, Hingerty BE, Singh SB, et al. Solution conformation of the major adduct between the carcinogen (+)-*anti*-benzo[*a*]pyrene diol epoxide and DNA. Proc Natl Acad Sci U S A. 1992;89: 1914-1918.
2. Cosman M, Hingerty BE, Luneva N, Amin S, Geacintov NE, et al. Solution conformation of the (-)-*cis*-*anti*-benzo[*a*]pyrenyl-dG adduct opposite dC in a DNA duplex: intercalation of the covalently attached BP ring into the helix with base displacement of the modified deoxyguanosine into the major groove. Biochemistry. 1996;35: 9850-9863.
3. Case DA, Darden TA, Cheatham III TE, Simmerling CL, Wang J, et al. (2006) AMBER 9. San Francisco: University of California.
4. Ryckaert JP, Ciccotti G, Berendsen HJC. Numerical-Integration of Cartesian Equations of Motion of a System with Constraints - Molecular-Dynamics of N-Alkanes. J Comput Phys. 1977;23: 327-341.
5. Harvey SC, Tan RKZ, Cheatham TE. The flying ice cube: Velocity rescaling in molecular dynamics leads to violation of energy equipartition. J Comput Chem. 1998;19: 726-740.
6. Darden T, York D, Pedersen L. Particle Mesh Ewald - an N.Log(N) Method for Ewald Sums in Large Systems. J Chem Phys. 1993;98: 10089-10092.
7. Essmann U, Perera L, Berkowitz ML, Darden T, Lee H, et al. A Smooth Particle Mesh Ewald Method. J Chem Phys. 1995;103: 8577-8593.
8. Jorgensen WL, Chandrasekhar J, Madura JD, Impey RW, Klein ML. Comparison of Simple Potential Functions for Simulating Liquid Water. J Chem Phys. 1983;79: 926-935.
9. Berendsen HJC, Postma JPM, Vangunsteren WF, Dinola A, Haak JR. Molecular-Dynamics with Coupling to an External Bath. J Chem Phys. 1984;81: 3684-3690.
10. Yan SX, Shapiro R, Geacintov NE, Broyde S. Stereochemical, structural, and thermodynamic origins of stability differences between stereoisomeric benzo[*a*]pyrene diol epoxide deoxyadenosine adducts in a DNA mutational hot spot sequence. J Am Chem Soc. 2001;123: 7054-7066.
11. Mocquet V, Kropachev K, Kolbanovskiy M, Kolbanovskiy A, Tapias A, et al. The human DNA repair factor XPC-HR23B distinguishes stereoisomeric benzo[*a*]pyrenyl-DNA lesions. EMBO J. 2007;26: 2923-2932.
12. Frisch MJ, Trucks GW, Schlegel HB, Scuseria GE, Robb MA, et al. (2003) Gaussian 03. Wallingford CT: Gaussian, Inc.
13. Bayly CI, Cieplak P, Cornell WD, Kollman PA. A Well-Behaved Electrostatic Potential Based Method Using Charge Restraints for Deriving Atomic Charges - the Resp Model. J Phys Chem. 1993;97: 10269-10280.
14. Mol CD, Izumi T, Mitra S, Tainer JA. DNA-bound structures and mutants reveal abasic DNA binding by APE1 and DNA repair coordination [corrected]. Nature. 2000;403: 451-456.
15. Starostenko LV, Rechkunova NI, Lebedeva NA, Kolbanovskiy A, Geacintov NE, et al. Human DNA polymerases catalyze lesion bypass across benzo[a]pyrene-derived DNA adduct clustered with an abasic site. DNA Repair (Amst). 2014;24C: 1-9.
16. Berman HM, Westbrook J, Feng Z, Gilliland G, Bhat TN, et al. The Protein Data Bank. Nucleic Acids Res. 2000;28: 235-242.
