# Supplementary material for: Resistance to Nucleotide Excision Repair of Bulky Guanine Adducts Opposite Abasic Sites in DNA Duplexes and Relationships between Structure and Function
Source: PLoS One. 2015 Sep 4;10(9):e0137124. doi: 10.1371/journal.pone.0137124 (PMC4560436; doi:10.1371/journal.pone.0137124)
Supplement: S1 Table — (DOCX) [file pone.0137124.s006.docx]

Table S1: AMBER atom type, connection type, and partial charge assignments for the THF site.

| **Atom name** | **Atom type** | **Connection type** | **Partial charge** |
| --- | --- | --- | --- |
| P | P | M | 1.221651 |
| O1P | O2 | E | -0.79246 |
| O2P | O2 | E | -0.79246 |
| O5' | OS | M | -0.50872 |
| C5' | CT | M | 0.012571 |
| H5'1 | H1 | E | 0.074727 |
| H5'2 | H1 | E | 0.074727 |
| C4' | CT | M | 0.184592 |
| H4' | H1 | E | 0.106699 |
| O4' | OS | E | -0.42117 |
| C3' | CT | M | 0.018587 |
| H3' | H1 | E | 0.099447 |
| C2' | CT | 3 | -0.01712 |
| H2'1 | HC | E | 0.036084 |
| H2'2 | HC | E | 0.036084 |
| C1' | CT | B | 0.048616 |
| H1'1 | H2 | E | 0.074487 |
| H1'2 | H2 | E | 0.074487 |
| O3' | OS | M | -0.53083 |
