# Supplementary material for: Resistance to Nucleotide Excision Repair of Bulky Guanine Adducts Opposite Abasic Sites in DNA Duplexes and Relationships between Structure and Function
Source: PLoS One. 2015 Sep 4;10(9):e0137124. doi: 10.1371/journal.pone.0137124 (PMC4560436; doi:10.1371/journal.pone.0137124)
Supplement: S2 Table — (DOCX) [file pone.0137124.s007.docx]

Table S2: AMBER atom type, connection type, and partial charge assignments for the *trans*-B[*a*]P-dG adduct.

| **Atom name** | **Atom type** | **Connection type** | **Partial charge** |
| --- | --- | --- | --- |
| P | P | M | 1.220933 |
| O1P | O2 | E | -0.79235 |
| O2P | O2 | E | -0.79235 |
| O5' | OS | M | -0.49903 |
| C5' | CT | M | -0.05433 |
| H5'1 | H1 | E | 0.111134 |
| H5'2 | H1 | E | 0.111134 |
| C4' | CT | M | 0.011886 |
| H4' | H1 | E | 0.098695 |
| O4' | OS | S | -0.18884 |
| C1' | CT | B | -0.03743 |
| H1' | H2 | E | 0.132367 |
| N9 | N* | B | 0.049191 |
| C8 | CK | S | 0.142575 |
| H8 | H5 | E | 0.174776 |
| C4 | CB | S | 0.013205 |
| C5 | CB | B | 0.312362 |
| N7 | NB | E | -0.64035 |
| C6 | C | B | 0.412122 |
| O6 | O | E | -0.55145 |
| N1 | NA | B | -0.30417 |
| H1 | H | E | 0.27978 |
| C2 | CA | B | 0.417552 |
| N3 | NC | E | -0.3907 |
| N | N2 | B | -0.46977 |
| HN | H | E | 0.304538 |
| CC10 | CT | B | 0.03351 |
| HC10 | H1 | E | 0.203809 |
| CC9 | CT | 3 | 0.078171 |
| HC9 | H1 | E | 0.097368 |
| O9 | OH | S | -0.65312 |
| HO9 | HO | E | 0.428533 |
| CC8 | CT | 3 | 0.100783 |
| HC8 | H1 | E | 0.164826 |
| O8 | OH | S | -0.65448 |
| HO8 | HO | E | 0.405272 |
| CC7 | CT | 3 | 0.171664 |
| HC7 | H1 | E | 0.082383 |
| O7 | OH | S | -0.63869 |
| HO7 | HO | E | 0.409503 |
| C61 | CA | S | -0.02855 |
| CC6 | CA | B | -0.18311 |
| HC6 | HA | E | 0.167616 |
| C51 | CA | B | 0.00876 |
| C123 | CA | E | 0.00218 |
| CC5 | CA | B | -0.13185 |
| HC5 | HA | E | 0.142132 |
| CC4 | CA | B | -0.21768 |
| HC4 | HA | E | 0.159215 |
| C31 | CA | B | 0.048755 |
| C122 | CA | E | 0.115736 |
| CC3 | CA | B | -0.17922 |
| HC3 | HA | E | 0.162085 |
| CC2 | CA | B | -0.21397 |
| HC2 | HA | E | 0.173083 |
| CC1 | CA | B | -0.15019 |
| HC1 | HA | E | 0.144085 |
| C121 | CA | S | -0.00123 |
| CC12 | CA | B | -0.14845 |
| HC12 | HA | E | 0.117305 |
| CC11 | CA | B | -0.02987 |
| HC11 | HA | E | -0.02657 |
| C102 | CA | S | -0.07289 |
| C101 | CA | E | 0.024121 |
| C3' | CT | M | 0.095394 |
| H3' | H1 | E | 0.152067 |
| C2' | CT | B | 0.034181 |
| H2'1 | HC | E | 0.037602 |
| H2'2 | HC | E | 0.037602 |
| O3' | OS | M | -0.53935 |
