# Supplementary material for: Resistance to Nucleotide Excision Repair of Bulky Guanine Adducts Opposite Abasic Sites in DNA Duplexes and Relationships between Structure and Function
Source: PLoS One. 2015 Sep 4;10(9):e0137124. doi: 10.1371/journal.pone.0137124 (PMC4560436; doi:10.1371/journal.pone.0137124)
Supplement: S3 Table — (DOCX) [file pone.0137124.s008.docx]

Table S3. Chemical Shifts (ppm) of the *trans*-B[*a*]P-dG:AB 11mer Duplex in D2O buffer, pH 6.8 at 10 ºC.

| Nucleotide | H8/H6 | Me/H5 | H1’ | H2’ | H2” | H3’ | H4’ | H5’/H5” | -NH2, NH or H2 |
| --- | --- | --- | --- | --- | --- | --- | --- | --- | --- |
| C1 | 7.49 | 5.66 | 5.69 | 2.23 | 1.78 | 4.40 | 3.85 | 3.50/3.47 |  |
| C2 | 7.34 | 5.43 | 5.03 | 2.12 | 1.92 | 4.57 | 3.83 | 3.77/3.72 | 8.25/6.71 |
| A3 | 8.09 |  | 5.98 | 2.57 | 2.46 | 4.76 | 4.16 | 3.76/3.84 | 7.51 |
| T4 | 6.67 | 1.00 | 5.41 | 1.59 | 1.23 | 4.42 | 3.97 |  | 13.25 |
| C5 | 5.96 |  | 5.25 | 1.46 | 0.77 | 3.97 | 3.73 |  | 7.18/6.61 |
| G6* | 7.96 |  | 6.49 | 3.02 | 2.64 | 5.13 | 4.48 | 3.96/3.80 |  |
| C7 | 7.34 | 5.45 | 4.25 |  | 1.66 | 4.35 | 3.72 |  | 7.15/6.61 |
| T8 | 7.19 | 1.23 | 5.36 | 2.24 | 1.91 | 4.57 |  |  | 13.11 |
| A9 | 7.96 |  | 5.89 | 2.56 | 2.38 | 4.48 | 4.12 | 4.01/3.90 | 7.05 |
| C10 | 7.08 | 5.04 | 5.62 | 2.14 | 1.79 | 4.49 |  |  | 7.91/6.46 |
| C11 | 7.31 | 5.35 | 5.92 | 1.97 |  | 4.27 | 3.75 |  |  |
|  |  |  |  |  |  |  |  |  |  |
| G12 | 7.64 |  | 5.48 | 2.47 | 2.38 | 4.56 | 3.44 | 3.94/3.89 |  |
| G13 | 7.55 |  | 5.78 | 2.56 | 2.39 |  |  |  | 12.54 |
| T14 | 7.06 | 1.17 | 5.49 | 2.27 | 1.95 | 4.65 | 3.98 |  | 13.29 |
| A15 | 7.89 |  | 5.84 | 2.68 | 2.42 | 4.82 | 4.16 |  | 6.85 |
| G16 | 7.50 |  | 5.69 | 2.52 | 2.37 | 4.80 | 4.16 | 3.98/3.72 | 11.02 |
| TF17 |  |  |  | 1.66 | 1.78 |  |  |  |  |
| G18 | 7.87 |  | 5.03 | 2.49 |  | 4.35 | 3.98 | 3.71/ | 11.09 |
| A19 | 8.17 |  | 6.04 | 2.70 | 2.57 | 4.85 | 4.27 | 3.98/3.87 | 7.49 |
| T20 | 6.88 | 1.19 | 5.32 | 1.91 | 1.57 | 4.55 | 4.00 | 3.84/ |  |
| G21 | 7.57 |  | 5.33 | 2.44 | 2.40 | 4.39 | 4.08 | 3.94/3.74 | 12.67 |
| G22 | 7.55 |  | 5.87 | 2.27 | 2.08 |  |  |  |  |
