# Supplementary material for: Resistance to Nucleotide Excision Repair of Bulky Guanine Adducts Opposite Abasic Sites in DNA Duplexes and Relationships between Structure and Function
Source: PLoS One. 2015 Sep 4;10(9):e0137124. doi: 10.1371/journal.pone.0137124 (PMC4560436; doi:10.1371/journal.pone.0137124)
Supplement: S4 Table — (DOCX) [file pone.0137124.s009.docx]

Table S4. Nucleic acid chemical shifts of the 5’-CCATCGCTACC-3’• 5’-GGTAGCGATGG-3’ 11 mer unmodified duplex (Unpublished data, kindly provided by Dr. Monique Cosman).

|  | H1' | H2' | H2" | H3' | H4' | H6/H8 | H2/H5/Me | Imino& amino* |
| --- | --- | --- | --- | --- | --- | --- | --- | --- |
| C1 | 5.95 | 2.07 | 2.50 | 4.64 | 4.08 | 7.72 | 5.91 | 7.93*_b_*/6.93*_nb_* |
| C2 | 5.44 | 2.18 | 2.44 | 4.85 | 4.12 | 7.6 | 5.70 | 8.56*_b_*/6.94*_nb_* |
| A3 | 6.32 | 2.75 | 2.97 | 5.04 | 4.45 | 8.38 | 7.78 | N.A. |
| T4 | 5.87 | 2.01 | 2.43 | 4.83 | 4.17 | 7.15 | 1.41 | 13.58 |
| C5 | 5.65 | 2.00 | 2.38 | 4.75 | 4.1 | 7.4 | 5.54 | 8.38*_b_*/6.80*_nb_* |
| G6 | 5.85 | 2.61 | 2.7 | 4.94 | 4.33 | 7.86 |  | 12.83 |
| C7 | 5.83 | 2.42 | 2.63 | 4.69 | 4.15 | 7.32 | 5.27 | 8.16*_nb_*/6.55*_nb_* |
| T8 | 5.64 | 2.10 | 2.43 | 4.84 | 4.12 | 7.36 | 1.63 | 13.7 |
| A9 | 6.19 | 2.70 | 2.85 | 5.00 | 4.4 | 8.28 | 7.49 | N.A. |
| C10 | 5.91 | 2.04 | 2.41 | 4.81 | 4.18 | 7.32 | 5.38 | 8.20*_b_*/6.71*_nb_* |
| C11 | 6.18 | 2.22 | 2.53 | 4.51 | 4.00 | 7.59 | 5.67 | 8.17*_nb_*/7.0*_nb_* |
|  |  |  |  |  |  |  |  |  |
| G12 | 5.67 | 2.53 | 2.65 | 4.97 | 4.31 | 7.84 | ---- | N.A. |
| G13 | 5.99 | 2.63 | 2.78 | 4.95 | 4.38 | 7.82 | ---- | 12.83 |
| T14 | 5.63 | 2.10 | 2.43 | 4.86 | 4.19 | 7.26 | 1.43 | 13.58 |
| A15 | 6.05 | 2.70 | 2.89 | 5.02 | 4.39 | 8.17 | 7.37 | N.A. |
| G16 | 5.67 | 2.45 | 2.58 | 4.94 | 4.35 | 7.63 | ---- | 12.74 |
| C17 | 5.58 | 1.83 | 2.28 | 4.79 | 4.08 | 7.18 | 5.17 | 8.15*_b_*/6.28*_nb_* |
| G18 | 5.61 | 2.58 | 2.79 | 4.93 | 4.35 | 7.85 | ---- | 12.67 |
| A19 | 6.18 | 2.58 | 2.87 | 4.99 | 4.42 | 8.15 | 7.75 | N.A. |
| T20 | 5.65 | 1.76 | 2.17 | 4.79 | 4.07 | 7.02 | 1.37 | 13.68 |
| G21 | 5.62 | 2.51 | 2.61 | 4.98 | 4.34 | 7.85 | ---- | 12.89 |
| G22 | 6.12 | 2.48 | 2.34 | 4.63 | 4.19 | 7.76 | ---- | N.A. |

*Assigned from water NOESY.

'b' and 'nb' define hydrogen-bonded and non-hydrogen-bonded amino protons. Imino proton: no label.

N.A.: Unable to assign.
