# Supplementary material for: Resistance to Nucleotide Excision Repair of Bulky Guanine Adducts Opposite Abasic Sites in DNA Duplexes and Relationships between Structure and Function
Source: PLoS One. 2015 Sep 4;10(9):e0137124. doi: 10.1371/journal.pone.0137124 (PMC4560436; doi:10.1371/journal.pone.0137124)
Supplement: S5 Table — (DOCX) [file pone.0137124.s010.docx]

Table S5. Average values of hydrogen bond angles.

| C5:G18 | 146.5 (3.3) | 146.0 (15.6) | 112.4 (9.4) |
| --- | --- | --- | --- |
| C7:G16 | 137.0 (2.6) | 145.7 (8.4) | 136.0 (18.6) |
